# *Mllt10* knockout mouse model reveals critical role of Af10-dependent H3K79 methylation in midfacial development

**DOI:** 10.1038/s41598-017-11745-5

**Published:** 2017-09-20

**Authors:** Honami Ogoh, Kazutsune Yamagata, Tomomi Nakao, Lisa L. Sandell, Ayaka Yamamoto, Aiko Yamashita, Naomi Tanga, Mai Suzuki, Takaya Abe, Issay Kitabayashi, Toshio Watanabe, Daisuke Sakai

**Affiliations:** 1 Department of Biological Science, Graduate School of Humanities and Science, Nara Women’s University, Nara, Japan; 2 Division of Hematological Malignancy, National Cancer Center Research Institute, Tokyo, Japan; 3 Department of Oral Immunology and Infectious Diseases, University of Louisville, School of Dentistry, Louisville, KY USA; 4 Animal Resource Development Unit and Genetic Engineering Team, RIKEN Center for Life Science Technologies, Kobe, Japan; 5 Laboratory of Developmental Neurobiology, Graduate School of Brain Science, Doshisha University, Kyoto, Japan

## Abstract

Epigenetic regulation is required to ensure the precise spatial and temporal pattern of gene expression that is necessary for embryonic development. Although the roles of some epigenetic modifications in embryonic development have been investigated in depth, the role of methylation at lysine 79 (H3K79me) is poorly understood. Dot1L, a unique methyltransferase for H3K79, forms complexes with distinct sets of co-factors. To further understand the role of H3K79me in embryogenesis, we generated a mouse knockout of *Mllt10*, the gene encoding Af10, one Dot1L complex co-factor. We find homozygous *Mllt10* knockout mutants (*Mllt10*-KO) exhibit midline facial cleft. The midfacial defects of *Mllt10*-KO embryos correspond to hyperterolism and are associated with reduced proliferation of mesenchyme in developing nasal processes and adjacent tissue. We demonstrate that H3K79me level is significantly decreased in nasal processes of *Mllt10*-KO embryos. Importantly, we find that expression of *AP2α*, a gene critical for midfacial development, is directly regulated by Af10-dependent H3K79me, and expression *AP2α* is reduced specifically in nasal processes of *Mllt10*-KO embryos. Suppression of H3K79me completely mimicked the *Mllt10*-KO phenotype. Together these data are the first to demonstrate that Af10-dependent H3K79me is essential for development of nasal processes and adjacent tissues, and consequent midfacial formation.

## Introduction

Epigenetic modifications are defined as mechanisms that regulate gene expression without changing of DNA sequences^1^. Epigenetic modifications are not rigid throughout life of an organism, but are subject to change both in prenatal and postnatal stages of life^2–4^. In embryonic development, epigenetic changes are indispensable for normal development and perturbations of the normal epigenetic profile can cause birth defects. It is now known that environmental conditions in the uterus can impact epigenetic regulation in developing embryos. Maternal life style factors, such as diet, exercise, and medication, can disturb epigenetic signaling in embryos, causing, or increasing the severity of, congenital anomalies in offspring^5^.

Epigenetic modifications include DNA methylation, histone methylation, and histone acetylation. Additional epigenetic modifications include binding of Polycomb repressive complex, ATP-dependent chromatin remodeling complex, or noncoding RNAs. One of the most extensively studied epigenetic modifications is methylation of histone H3 at lysine residues^6^. Lysine residues in histones can be mono-, di-, or tri-methylated by two different kinds of methyltransferases: the SET-domain-containing protein, and Dot1/Dot1L^7,8^. The SET-domain-containing protein primarily mediates methylation of lysine residues at the 4th, 9th, 27th and 36th positions. In contrast, Dot1/DOT1L methyltransferase is unique for methylation of lysine residues located at position 79^9–12^. Methylation of histone H3 at lysine 79 (H3K79me) is distinct from other histone H3 methylations in that H3K79me is generally associated with transcriptional activation and elongation, while other methylations at other histone H3 residues can cause either activation of gene expression, or repression of gene expression, depending on the context^13,14^.

Recent studies have identified many important biological functions of *Dot1L*-mediated H3K79me. For example, perturbations in *DOT1L* have been implicated in the pathogenesis of mixed lineage leukemia (MLL)-rearranged leukemia and other cancers^15–19^. Inhibition of DOT1L increases reprogramming efficiency in the context of generating human iPS cells from dermal fibroblasts by introduction of Yamanaka factors^20^. During mouse embryonic development, *Dot1L* is ubiquitously expressed at early stages, and is required for yolk sac angiogenesis, hematopoiesis and vascular remodeling. *Dot1L* homozygous knockout embryos exhibit multiple morphological abnormalities associated with defects in hematovascular development, including growth retardation, enlarged hearts, and stunted tails. These defects lead to embryo death, usually by embryonic day 10.5 (E10.5)^21^. These studies demonstrate the importance Dot1L-mediated H3K79me in cancer and early stages of embryogenesis. However, because homozygous knockout of Dot1L results in embryonic lethality by E10.5, the role of H3K79me at later stages of embryogenesis has not been elucidated.

DOT1L forms large multiprotein complexes, which are composed of different combinations of co-factors^13^. The EAP core complex contains DOT1L, AF4, ENL and P-TEFb^22^. In contrast, DotCom is composed of DOT1L, AF9, AF10, ENL, AF17, TRRAP, Skp1 and β-catenin^23^. A third complex, identified by Bitoun *et al*., contains DOT1L, AF4, AF9, AF10, ENL and P-TEFb^24^. Each complex activates the expression of distinct target genes. For example, EAP core complex regulates *HOXA9* and *MEIS2* expression, and defects in this complex are associated with leukemogenesis^25^. The DotCom complex specifically regulates expression of Wingless/Wnt target genes through H3K79me^23^. These findings imply that specific combinations of co-factors present in DOT1L complexes define which target genes are regulated through H3K79me. Among those co-factors, biochemical interactions between AF10 and DOT1L have been studied extensively. Af10 protein has two important domains, a chromatin-associated PHD finger-Zn knuckle-PHD finger (PZP) domain, and an octapeptide motif and leucine zipper (OM-LZ) domain. AF10 binds directly to DOT1L through the OM-LZ domain^26^. Bound to DOT1L, AF10 simultaneously senses unmodified H3K27 via its PZP domain and enables DOT1L to methylate histone H3 at K79^26^. Although the biochemical mechanism of AF10 regulation of DOT1L activity has been defined in great detail, the biological function of Af10 *in vivo* has never been studied, and the role of Af10-dependent H3K79 methylation in embryonic development has not been examined.

To elucidate the role of H3K79me in embryonic development, we have examined the distribution and role of the Af10-encoding gene *Mllt10* in developing mouse embryos. We discovered that *Mllt10* is intensely expressed in developing facial primordia. Consistent with this expression pattern, *Mllt10*-KO mutant embryos exhibited severe midfacial abnormalities, including midline facial cleft and hypertelorism. We found that lack of *Mllt10* resulted in decreased mesenchymal cell proliferation in nasal processes and adjacent facial tissues of mutant embryos, suggesting pathogenic mechanism for the observed hypertelorism and midline facial cleft. *Mllt10*-KO embryos, lacking Af10, had reduced H3K79me in developing nasal processes and reduced expression of *AP2α*, a gene required for cranial neural crest cell (CNCC) development. Importantly, we demonstrated that the *AP2α* locus was enriched Af10-dependent H3K79me2, indicating that the influence of Af10-dependent H3K79me on *AP2α* gene expression is direct. Finally, we found that suppression of H3K79me by treatment with a small molecule inhibitor of Dot1L could mimic *Mllt10*-KO phenotypes, reducing cell proliferation and *AP2α* expression in nasal processes, and producing midline facial cleft defects. These results demonstrate that *Mllt10*-dependent H3K79me is critically required for nasal process morphogenesis and subsequent midfacial development. Our findings help to understand the cellular basis of midfacial development and, conversely, the pathogenesis of midline facial clefts such as occur of frontonasal dysplasia (FND; OMIM 136760, 613451, 613456)^27^.

## Results

### *Mllt10* is expressed in a tissue-specific manner in mouse embryos

Because complete loss of Dot1L-mediated H3K79me is lethal to an embryo at mouse stage E10.5^21^, investigation of the function of H3K79me at later stages by complete knockout of Dot1L is not possible. However, the importance of H3K79me can be studied by disrupting the function of specific cofactors that spatiotemporally determine Dot1L activity. One such critical cofactor of Dot1L is Af10. A mouse knockout of the Af10-encoding gene *Mllt10* has recently been generated and studied in the context of leukemia pathogenesis in adult bone marrow cells^28^. However, the function of *Mllt10* during prenatal development has not been investigated. In order to elucidate the role of Dot1L-mediated H3K79me during embryogenesis we have examined the effect of loss-of-function of the Dot1L cofactor Af10.

**Figure 1:**
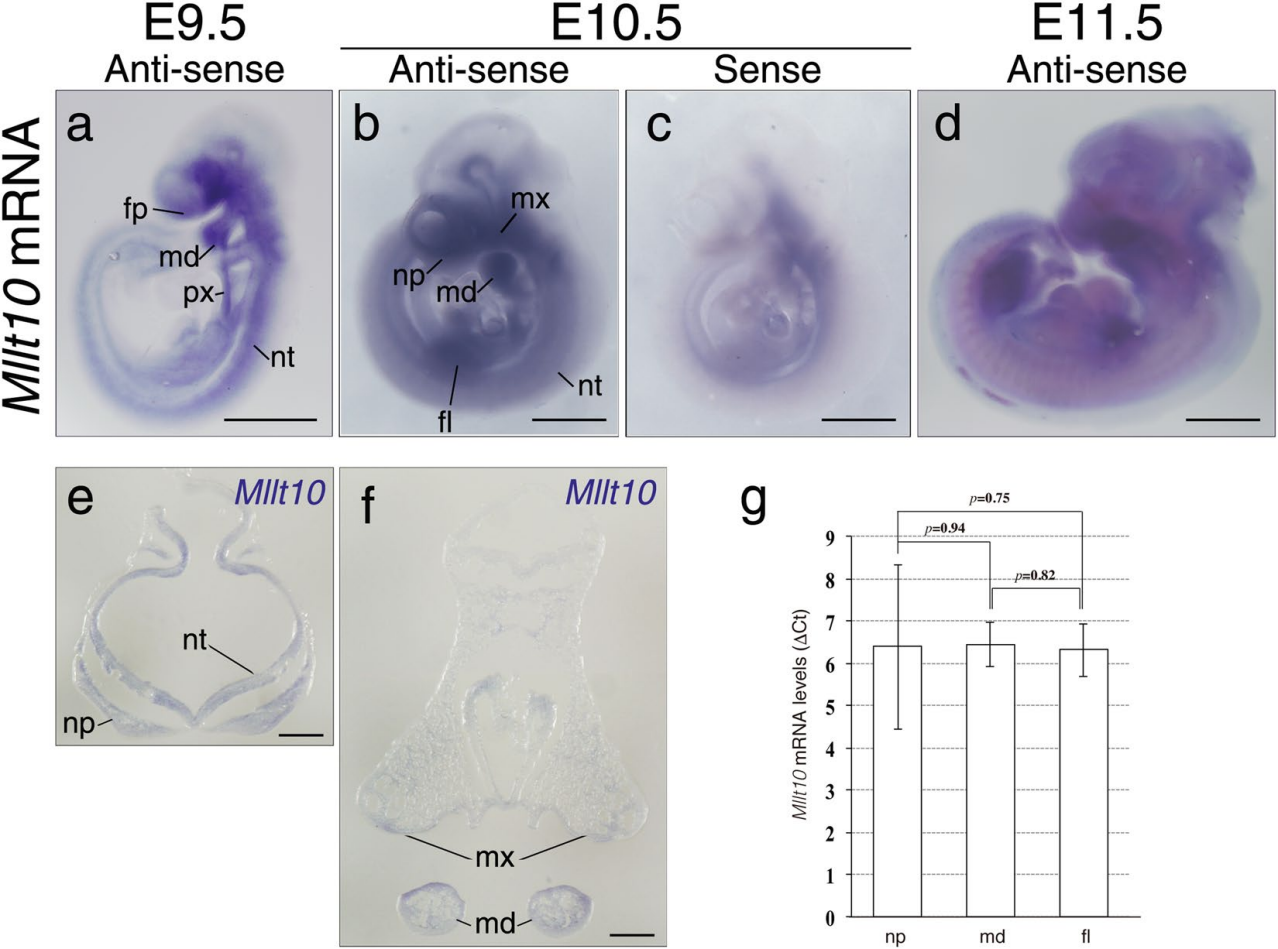
Expression pattern of *Mllt10* mRNA in mouse embryos. Expression of *Mllt10* mRNA was detected by whole-mount *in situ* hybridization of E9.5 (**a**), E10.5 (**b**) and E11.5 (**d**). (**c**) Non-specific signal detected by sense probe is shown. The low signal suggests specificity of *Mllt10* antisense probe in other panels. In each case 3–7 embryos were analyzed. Scale bars; 1 mm. Frontal section of E10.0 embryo stained with antisense probe for *Mllt10* at the level of the nasal process (**e**), and the level of the mandibular and maxillary process (**f**) are shown. Section images are representative of 2 embryonic specimens analyzed. Scale bars; 100 μm. fl; forelimb bud, fp; frontonasal process, md; mandibular arch, mx; maxillary process, np; nasal process, nt; neural tube, px; pharynx. (**g**) Expression level of *Mllt10* was quantified by RT-qPCR. Quantities of *Mllt10* mRNA were normalized to *Gapdh* mRNA and the ΔCT (CT value of the samples normalized to that of *Gapdh*) are presented as bar graph. Data are mean ± s.e. of 5 independent experiments. Statistical differences were assessed with Student’s *t*-test, and *p*-values are shown.

Although Gray *et al*. have examined the expression of *Mllt10* in embryonic brain^29^, the pattern of expression of *Mllt10* in other embryonic tissues is unknown. In order to begin to elucidate the role of Af10 during normal embryogenesis, we firstly examined the expression pattern of *Mllt10* mRNA in wild-type mouse embryos by whole-mount *in situ* hybridization. At E9.5, *Mllt10* was strongly expressed in facial primordia, including the frontonasal process, mandibular processes, neural tube, and pharynx (Fig. 1a). At E10.5, *Mllt10* mRNA was detected at very low level in all regions of the embryo, with domains of stronger intensity in nasal processes, mandibular processes, maxillary processes, neural tube, and early forelimb buds (Fig. 1b). *Mllt10* sense probe did not detect signal in facial primordia or forelimb buds (Fig. 1c), demonstrating that antisense *Mllt10* probes correctly detected *Mllt10* mRNA expression. At E11.5, *Mllt10* mRNA was expressed ubiquitously throughout the embryo at low levels, with domains of relative intense expression in facial primordia and limb buds (Fig. 1d). In order to determine the details of *Mllt10* expression in the craniofacial tissues, we further sectioned embryos stained for *Mllt10* mRNA. *Mllt10* mRNA was detected nasal processes, neural tube, maxillary processes, and mandibular processes (Fig. 1e,f). Within these regions *Mllt10* mRNA is detected in both mesenchyme and epithelium, including olfactory epithelium. These data demonstrate that Af10 is present in a spatiotemporally restricted pattern in developing embryos, being expressed particularly in primordia of the developing face, jaw, and limbs. We further validated *Mllt10* mRNA expression in nasal processes, mandibular processes and forelimb buds of E10.5 embryos by RT-qPCR. *Mllt10* mRNA expression level was nearly equivalent in each of those tissues (Fig. 1g).

### Generation of *Mllt10* knockout mice

In order to address the biological function of *Mllt10* in embryonic development, we generated a new conditional mouse knockout allele of *Mllt10*. Two domains are critical for Af10 function, the PZP domain, and OM-LZ domain. The OM-LZ domain is required for direct binding with Dot1L and subsequent H3K79me activity^30^. Moreover, as demonstrated by oncogenic activity of the human leukemia fusion gene *MLL-MLLT10*, the OM-LZ domain is important for target gene expression^31^. We therefore designed a conditional cassette with the aim of disrupting the Af10 OM-LZ domain, which is encoded by *Mllt10* exon 16 and exon 17. Targeting of the conditional construct produces an allele of *Mllt10* with exon 16 flanked by *loxP* sites followed by an *frt*-flanked Neo cassette (*Mllt10* ^*flox*^) (Supplementary Fig. S1b). ES cells containing the conditional *Mllt10* knockout allele were generated as described in Methods and Supplementary Fig. S1a–c. Targeted ES cells were injected into 8-cell stage embryos to obtain chimeric mice.

Chimeric mice bearing the *Mllt10* conditional construct were crossed with C57BL/6 mice to obtain mice heterozygous for the conditional *Mllt10* ^*flox*^ allele (*Mllt10* ^*flox/*+^). *Mllt10* ^*flox/*+^ mice were crossed with a ubiquitous Cre deleter strain (CAG-Cre TG) to induce deletion of exon16 (Supplementary Fig. S1d), thereby generating mice heterozygous for *Mllt10* exon16-deletion (*Mllt10*-Het). *Mllt10*-Het mice were viable, fertile and displayed no gross abnormalities. *Mllt10*-Het mice were intercrossed to generate homozygous *Mllt10* exon16-deletion (*Mllt10*-KO) mutant embryos. To verify correct targeting of the *Mllt10* locus, and to confirm elimination of *Mllt10* exon 16, Southern blot analysis was performed using probes hybridizing outside the homology arms of the targeting construct. To examine DNA from embryos, we initially cultured mouse embryonic fibroblast (MEF) cells from wild-type, *Mllt10*-Het and *Mllt10*-KO embryos, respectively. Southern blot analysis confirmed correct targeting (Supplementary Fig. S1f,g). Deletion of exon 16 was confirmed by PCR genotyping (Supplementary Fig. S1h). Additionally, we performed western blot analysis to determine whether or not Af10 protein is eliminated in *Mllt10*-KO mutant cells. Intact full-length Af10 protein was almost completely absent in MEF cells from *Mllt10*-KO mutant embryos (Supplementary Fig. S1i). A very faint band corresponding to a C-terminal truncation form of Af10 protein was detected (Supplementary Fig. S1i), however the amount of the truncated peptide was very small, and the activity of the peptide is likely to be negligible. We therefore interpret that *Mllt10* exon16-deletion allele is a null mutation. We used *Mllt10*-Het mice without Neo cassette to obtain *Mllt10*-KO pups (Supplementary Fig. S1e).

Intercrossing of *Mllt10*-Het mice did not produce any *Mllt10*-KO viable pups, indicating that complete loss of Af10 is embryonic lethal prior to birth. To determine the stage of embryonic lethality, embryos from *Mllt10*-Het intercrosses were genotyped at E10.5, E13.5 and E16.5. Relative to litters examined at E10.5, litters examined at E13.5 and E16.5, had reduced numbers of embryos per litter and placental debris and signs of resorptions in the uterus, indicating embryo death prior to E16.5. In rare cases, dead embryos exhibiting growth arrest and ischemia were obtained at E16.5 (Supplementary Fig. S1j; dead). Although homozygous *Mllt10*-KO embryos would be expected at a frequency of 25%, of forty-two live embryos obtained at E16.5, none were *Mllt10*-KO (Table 1). These data demonstrate that *Mllt10*-KO embryos die before E16.5. At E13.5, many living *Mllt10*-KO embryos were observed, although the embryos were abnormal with dorsal swelling due to severe hemorrhage and edema (Supplementary Fig. S1k). Among 70 embryos obtained at E13.5, 19 (27.1%) were wild-type, 37 (52.9%) were *Mllt10*-Het and 14 (20.0%) were *Mllt10*-KO at E13.5 (Table 1). The lower than expected number of *Mllt10*-KO embryos suggests that some mutant embryos die prior to E13.5. At E10.5, wild-type, *Mllt10*-Het and *Mllt10*-KO embryos were obtained at the expected ratio of approximately 1:2:1 (Table 1), demonstrating that *Mllt10*-KO lethality occurs after E10.5. These results show that *Mllt10*-KO embryos start to die around E13.5 due to vascular defects.

**Table 1:** Genotype analysis of *Mllt10*-Het intercross progeny. WT, wild-type; Het, *Mllt10*-Het; KO, *Mllt10*-KO.

Stage	total	WT	Het	KO(%)
E16.5	42	21 (50.0)	21 (50.0)	0 (0)
E13.5	70	19 (27.1)	37 (52.9)	14 (20.0)
E10.5	101	20 (19.8)	56 (55.4)	25 (24.8)

### *Mllt10-KO* embryos exhibit severe midline facial cleft and hypertelorism

**Figure 2:**
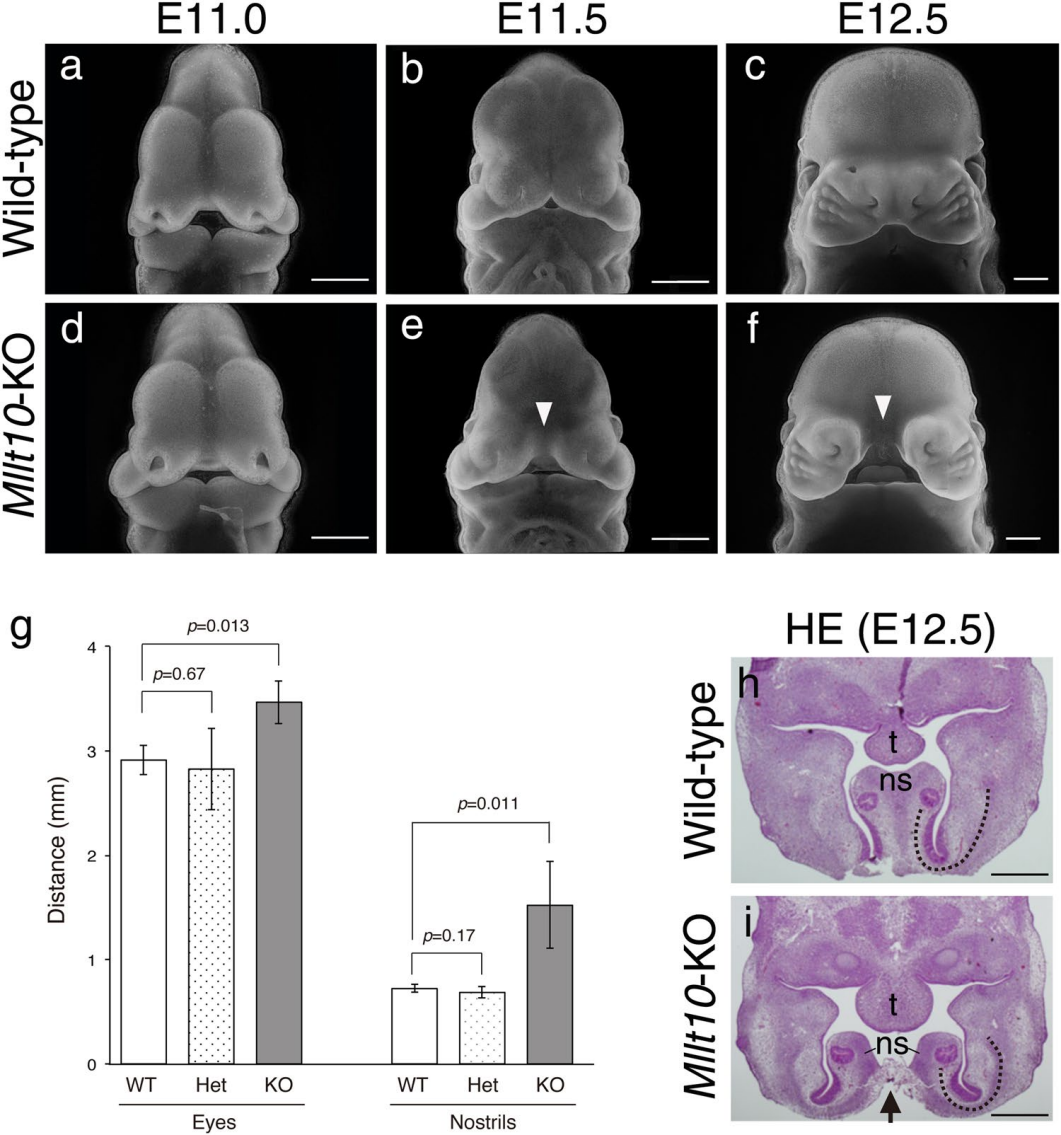
*Mllt10*-KO embryos exhibit severe midline facial cleft and hypertelorism. (**a**–**c**) Normal craniofacial development of wild-type embryos during E11.0 to E12.5. (**d**–**f**) Abnormal craniofacial development of *Mllt10*-KO embryos, includes midline facial cleft (arrowheads). Scale bars; 500 μm. (**g**) The distance between the eyes and between the nostrils was measured. WT; wild-type, Het; *Mllt10*-Het, KO; *Mllt10*-KO. Data are mean ± s.e. of 5 embryos. Statistical differences were assessed with Student’s *t*-test, and *p*-values are shown. (**h** and **i**) Hematoxylin/Eosin staining of transverse sections at nasal level are shown. Splitting of nasal septum is indicated by arrow. Curvature of the nasal processes is indicated by dotted-line. ns; nasal septum, t; tongue. Scale bars; 500 μm.

The strong expression *Mllt10* in facial primordia and limb buds suggests that *Mllt10* plays a role in development of these structures. Although *Mllt10* is expressed in developing mandible and limbs, examination of *Mllt10*-KO embryos at E12.5 revealed no gross morphological defects in these structures (Supplementary Figs S2 and S3). In contrast, striking morphological defects were observed in the developing snouts of E12.5 *Mllt10*-KO embryos, which exhibited obvious midline separation (Fig. 2c,f). These data demonstrate that lack of *Mllt10* causes a defect of nasal process development. In mutant embryos the gross morphology of nasal processes appeared to be normal until E11.0 (Fig. 2a,d). The first obvious morphological defect in facial morphogenesis in mutant embryos, a gap between medial nasal processes, became visible at E11.5 (Fig. 2b,e). The developing midline cleft in E12.5 *Mllt10*-KO embryos could be quantified as a significant increase in distance between each nostril (Fig. 2g; Nostrils). Transverse sections through maxillae at nasal level showed that nasal processes of *Mllt10* mutant embryos were reduced in size and slightly more curved toward the medial axis relative to wild type counterparts (Fig. 2h,i; dotted-line), but overall structural and anatomical features were generally normal. Interestingly, the precursor of nasal septum, which can be recognized by dense staining at the midline in wild type embryos, was completely split in two in *Mllt10*-KO embryos (Fig. 2i). Thus loss of *Mllt10* causes dramatic morphological midface developmental abnormalities, but the malformations do not result from defects in cell differentiation or tissue patterning in nasal processes.

Many midfacial defects are accompanied by the phenotype of ocular hypertelorism^32^, a term describing abnormally wide facial width, which can be measured as distance between the eyes. In addition to developing midline cleft, *Mllt10*-KO embryos exhibited an increased distance between each eye relative to wild type embryos or *Mllt10*-Het embryos, (Fig. 2g; Eyes). Ocular hypertelorism, the increased distance between eyes, is a symptom of expansion of midline structures.

### Loss of *Mllt10* reduces proliferation of cranial neural crest cells in nasal processes

**Figure 3:**
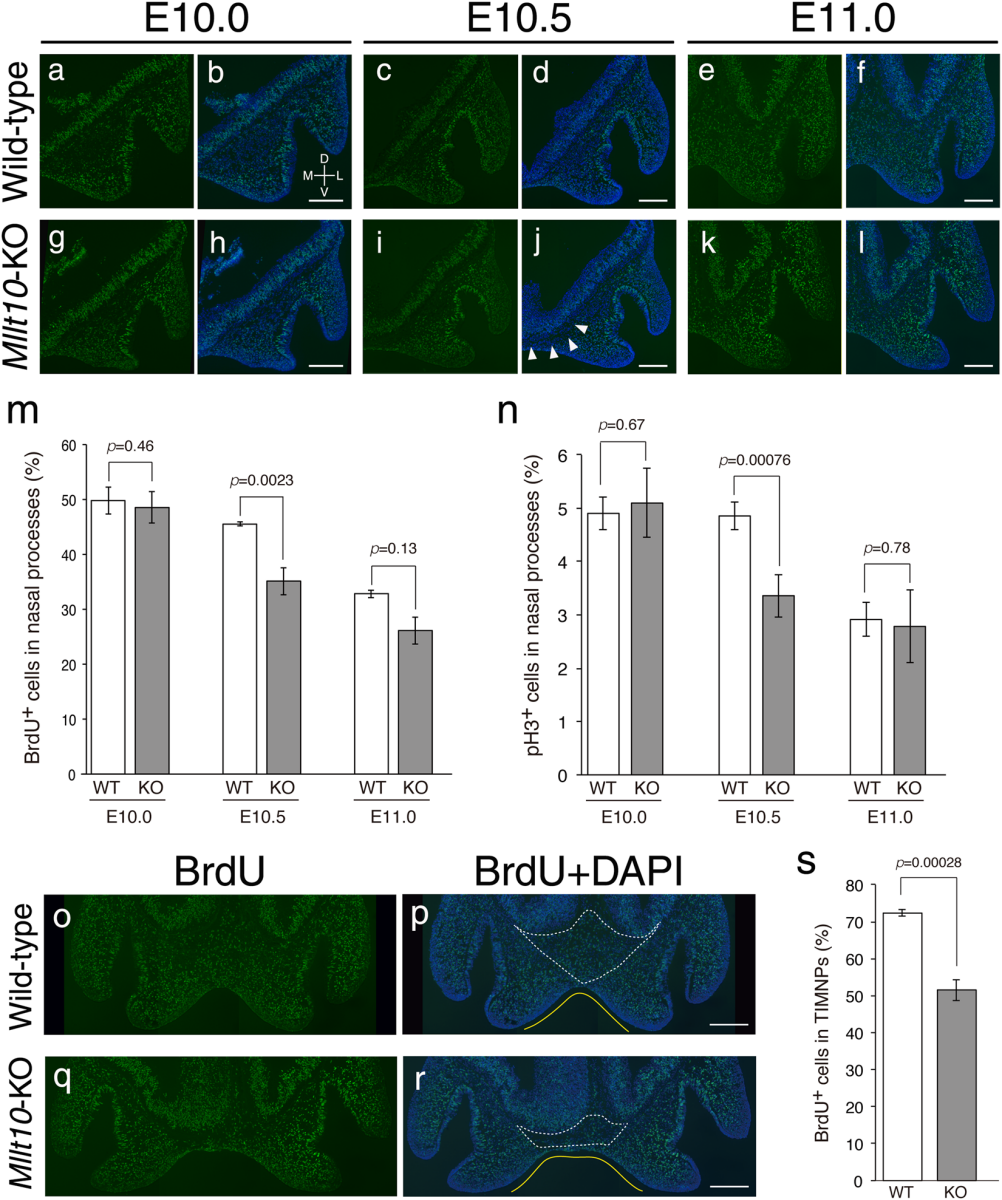
Proliferation defects in mesenchymal cells of *Mllt10*-KO embryo nasal processes. BrdU-positive proliferating cells in nasal process were detected in frontal sections of wild-type (**a**–**f**) and *Mllt10*-KO (**g**–**l**) embryo heads at indicated embryonic stages. *Mllt10*-KO embryos had a marked reduction of BrdU-positive cells at E10.5, indicated by arrowheads in (**j**). Scale bars; 100 μm. Directional planes for (**a–l**) were shown in (**b**) (D; dorsal, V; ventral, M; medial and L; lateral). Bar graph depicting average of numbers of BrdU-positive cells (**m**), and pH3-positive cells (**n**) in the nasal processes of E10.0, E10.5 and E11.0 wild-type (WT) and *Mllt10*-KO (KO) embryos. The number of BrdU-positive cells and pH3-positive cells were counted in both of medial and lateral nasal process on 3 histological sections obtained from 3 different embryos. Data are mean ± s.e. Statistical differences were assessed with Student’s *t*-test, and *p*-values are shown. (**o**–**r**) BrdU-positive proliferating cells in TIMNPs were detected using frontal sections of E11.0 wild-type and *Mllt10*-KO embryos. TIMNPs indicated by white dotted-lines. The angle made by opposing medial nasal processes indicated by yellow line. Scale bars; 100 μm. (**s**) The number of BrdU-positive cells was counted in TIMNPs on 3–4 histological sections obtained from 3 embryos. Data are mean ± s.e. Statistical differences were assessed with Student’s *t*-test, and *p*-values are shown.

Facial primordia are populated predominantly by cranial neural crest cells (CNCCs). CNCC proliferation drives expansion and elongation of individual facial processes, which grow and fuse to adjacent processes, eventually forming the complex structure of the face and jaw^33,34^. We hypothesized that midline facial cleft in *Mllt10*-KO embryos might be caused by reduced proliferation and/or survival of CNCCs. To test this hypothesis, cell proliferation in facial primordia of wild-type and *Mllt10*-KO embryos was examined by BrdU pulse labeling. BrdU was injected into pregnant dams intraperitoneally, and then embryos were collected after 1 hour. At E10.0, no significant difference was detected in proliferation of mesenchymal cells in nasal processes of *Mllt10*-KO embryos (Fig. 3g,h,m; 48.5 ± 2.5%) versus wild-type embryos (Fig. 3a,b,m; 49.7 ± 0.4%). In contrast, at E10.5, cell proliferation was significantly decreased in *Mllt10*-KO (Fig. 3i,j,m; 35.1 ± 2.5%) as compared with wild-type (Fig. 3c,d,m; 45.6 ± 0.7%) embryos. The reduction of cell proliferation at E10.5 was particularly prominent on the medial side of the medial nasal process near the midline of *Mllt10*-KO embryos (Fig. 3j; arrowheads). The reduction in proliferation was predominantly limited to the E10.5 stage of development. Although cell proliferation was slightly reduced in *Mllt10*-KO embryos (Fig. 3k,l,m; 26.1 ± 3.1%) as compared with wild-type (Fig. 3e,f,m; 32.8 ± 5.2%) at E11.0, the difference was not statistically significant at that stage. As an additional assay for cell proliferation, we also counted the number of phosphorylated-histone H3 (pH3)-positive mitotic cells in nasal processes. We found that, consistent with the reduction of synthesis-phase BrdU-positive cells at E10.5, pH3-positive mitotic cells were also decreased in *Mllt10*-KO embryos at that stage (Fig. 3n, Supplementary Fig. S4). In addition to quantifying cell proliferation, we also investigated whether there were differences in the amount of programmed cell death in *Mllt10*-KO mutant embryos versus wild type. No ectopic apoptosis was detected in *Mllt10*-KO embryos from E10.0 to E11.0 (Supplementary Fig. S5), demonstrating that the hypoplasia of nasal processes observed in mutant embryos is not a result of excessive apoptosis.

Abnormalities of the developing midface were particularly evident in the tissue intervening between medial nasal processes (TIMNPs). At E11.0, the amount of TIMNPs mesenchymal tissue was drastically reduced in the *Mllt10*-KO embryos relative to wild-type littermate embryos (Fig. 3r; enclosed by a dotted-line). Consistently, BrdU-positive cells were markedly decreased in TIMNPs of *Mllt10*-KO embryos (Fig. 3q,s). The reduction in TIMNPs cell proliferation appeared to be related to the reduction of cell proliferation in medial side of medial nasal process at the earlier E10.5 stage of development (Fig. 3j). The reduction in amount of TIMNPs tissue corresponded to an altered angle between the two medial nasal processes. In wild-type embryos the angle between the medial nasal processes was acute, while in *Mllt10*-KO embryos the angle was obtuse (Fig. 3p,r; yellow line). As the result of this deformation, the distance between each medial nasal process was appreciably increased in *Mllt10*-KO embryos. Collectively, these data show that *Mllt10* is required for proliferation of CNCCs in nasal processes and in the adjacent TIMNPs region during midfacial development, and, further, that lack of *Mllt10* causes a reduction in the mass of CNCCs in TIMNPs which consequently contributes to midline facial cleft.

### Loss of *Mllt10* increases the proliferation of neural progenitor cells

**Figure 4:**
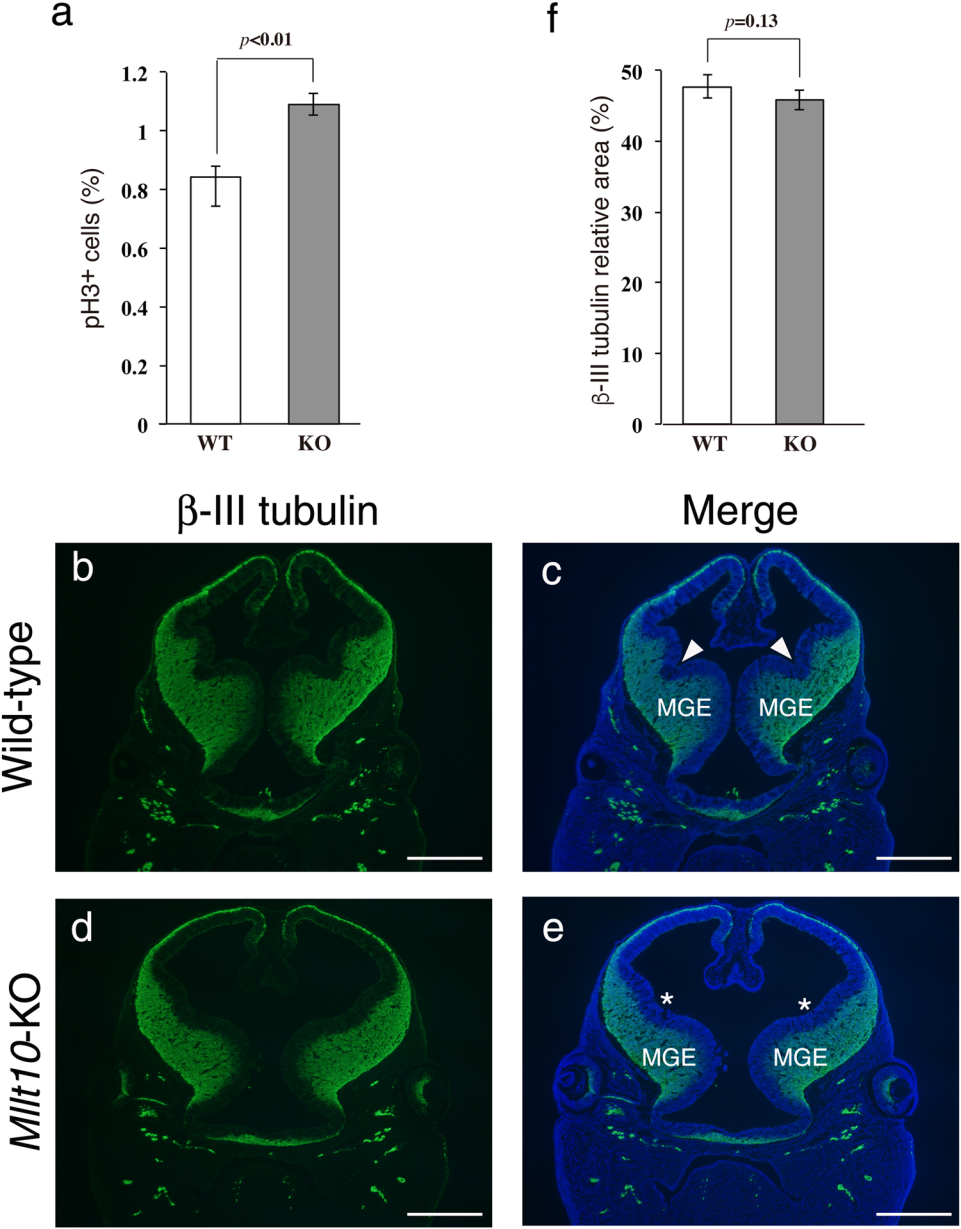
*Mllt10*-KO embryos exhibit abnormal forebrain development. (**a**) Bar graph depicting the percentage of pH3-positive cells in the forebrain of E12.5 wild-type (WT) and *Mllt10*-KO (KO) embryos. Neuron-specific β-III tubulin was immunostained in frontal sections of E12.5 wild-type (**b**,**c**) and *Mllt10*-KO (**d**,**e**) embryonic heads at eye level. The forebrain expanded laterally in *Mllt10*-KO embryos. Arrowheads indicate the boundary between lateral and medial ganglionic eminences. The boundary was obscure in *Mllt10*-KO forebrain (asterisks). MGE; medial ganglionic eminence. Scale bars; 500 μm. (**f**) Bar graph depicting relative area of immunostaining of anti-β-III tubulin antibody in the forebrain of E12.5 wild-type (WT) and *Mllt10*-KO (KO) embryos. The area of β-III tubulin-positive and the number of pH3-positive cells were analyzed in the forebrain on 3 histological sections obtained from 2 different embryos. Data are mean ± s.e. Statistical differences were assessed with Student’s *t*-test, and *p*-values are shown.

We next examined whether loss of *Mllt10* effected cell proliferation in tissues surrounding the mesenchyme of the nasal processes. The number of pH3-positive mitotic cells was not altered in olfactory epithelium, medial nasal process epithelium, lateral nasal process epithelium, mandibular epithelium, or mandibular mesenchyme of E10.5 *Mllt10*-KO embryos. Contrary to nasal process mesenchyme, which had a reduction in cell proliferation in mutant embryos, the neuroepithelium of E10.5 *Mllt10*-KO embryos had slightly increased cell proliferation (Supplementary Fig. S6a). At E12.5, the number of mitotic cells remained elevated in neuroepithelium of *Mllt10*-KO embryos as compared to wild-type (Fig. 4a). We further examined whether or not neurogenesis was affected in the forebrain of *Mllt10*-KO embryos by neuron-specific β-III tubulin immunostaining (Fig. 4b–e). The neuron layer was formed normally in the cortex in mutant embryos. Although neuronal development appeared to be normal, the forebrain of *Mllt10*-KO embryos was expanded, as manifested by lateral extension of ventral region of forebrain and increased distance between each medial ganglionic eminence (Fig. 4d,e). In addition, the boundary between lateral and medial ganglionic eminences was obscure in mutant embryos as compared to that of wild-type, it may be due to the expansion of the forebrain (Fig. 4c,e; asterisks). The number of neurons was estimated by measurement of the area positive for neuron-specific β-III tubulin. The number of neurons was not altered by loss of *Mllt10* (Fig. 4f). These results suggest that forebrain expansion is caused by abnormal proliferation of neural progenitor cells rather than aberrant neurogenesis. Collectively, the increased distance between eyes and expanded forebrain phenotypes suggest that hypertelorism contributes to midfacial defects in *Mllt10*-KO embryos.

### H3K79me2 is diminished by loss of *Mllt10*

**Figure 5:**
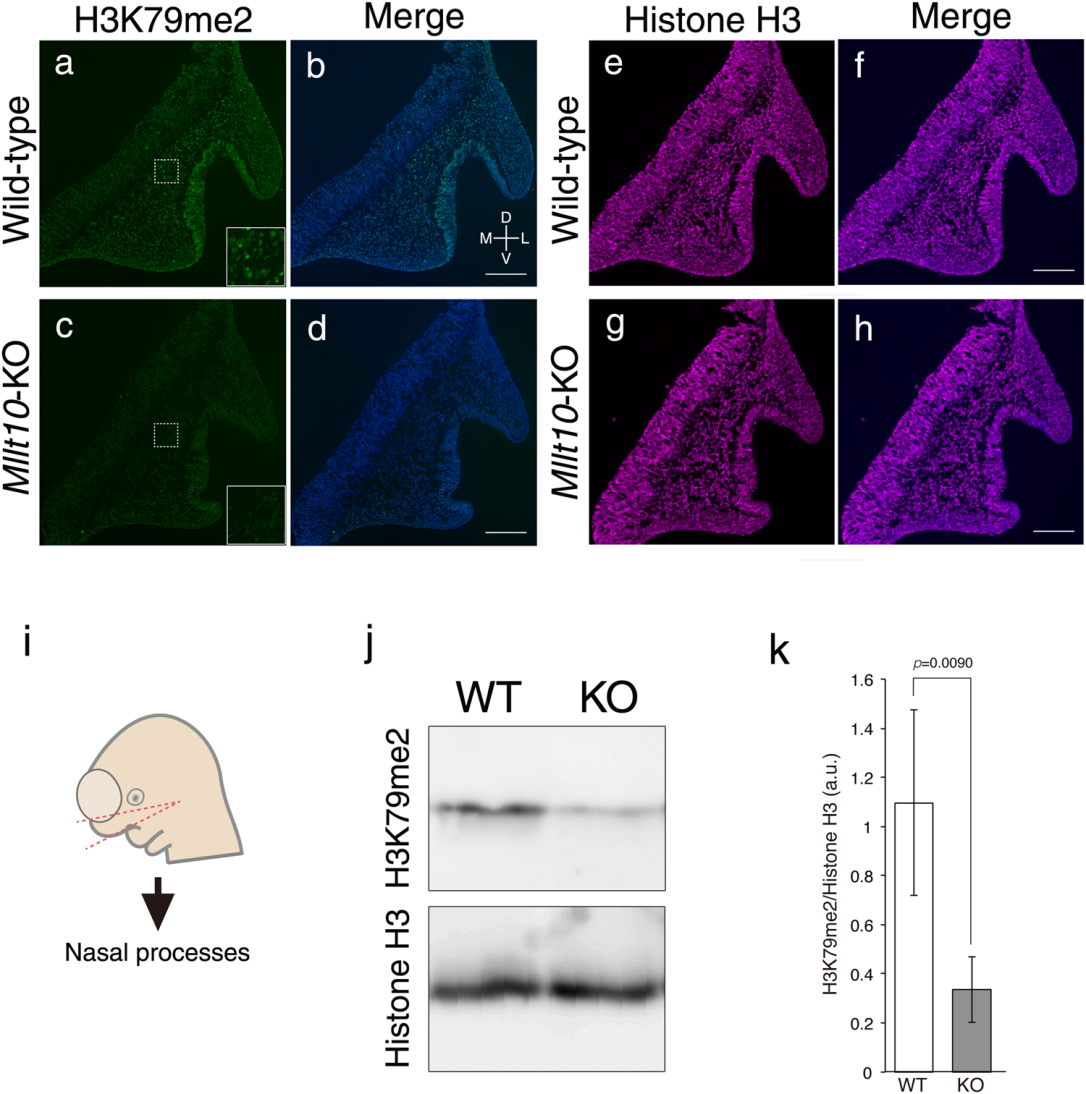
Loss of *Mllt10* leads to the decrease of H3K79me2 level. H3K79me2 was visualized by immunostaining using frontal sections of nasal processes of wild-type (**a** and **b**) and *Mllt10*-KO (**c** and **d**) embryos. Insets are magnifications of the region enclosed with dotted-line. 4–6 histological sections obtained from 6 embryos were analyzed. Scale bars; 100 μm. (**e**–**h**) Histone H3 expression was detected. Directional planes for (**a**–**h**) were shown in (**b**) (D; dorsal, V; ventral, M; medial and L; lateral). (**i**) Illustration of depicting dissection and isolation nasal process tissues from E10.5 embryo. Embryo head was cut along red dotted-line, and a piece of tissue including nasal processes, a small part of forebrain, and maxillary processes was collected for preparation of tissue lysate. (**j**) H3K79me2 level was analyzed by western blot using whole histones. Histone H3 was detected as internal control. The original image is presented in Supplementary Information. WT; wild-type, KO; *Mllt10*-KO. (**k**) Signals were assessed by densitometry and were normalized to Histone H3 signals. Quantification of individual signals is presented as bar graph. WT; wild-type, KO; *Mllt10*-KO. Data are mean ± s.e. of 3 experiments. Statistical differences were assessed with Student’s *t*-test, and *p*-value are shown.

In the context of leukemogenesis it has been demonstrated that Af10 functions as a co-factor of a Dot1L complex, specifically regulating Dot1L-mediated H3K79me and subsequent conversion from H3K79 monomethylation (H3K79me1) to dimethylation (H3K79me2)^26^. However, it remains unknown whether Af10 functions in the same manner to promote H3K79me2 during normal embryonic development. To examine the requirement for Af10 in H3K79me2 in developing embryos, we assayed H3K79me2 in wild-type embryos and *Mllt10*-KO embryos by immunostaining using an antibody specific for the H3K79me2 form of histone H3. In E10.5 wild-type embryos many H3K79me2-positive cells were seen within mesenchyme and epithelium of nasal processes, and in olfactory epithelium (Fig. 5a,b), demonstrating that H3K79me2 occurs during normal development. In contrast, very little or no H3K79me2 was detected in mesenchyme and epithelium of nasal process, or in olfactory epithelium, of *Mllt10*-KO embryos (Fig. 5c,d, Supplementary Fig. S6b,c,f,g). The diminished H3K79me2 signal was not caused by decreased expression of histone H3. Within the nasal process tissue, histone H3 was detected at comparable levels in *Mllt10*-KO and wild-type embryos (Fig. 5e–h). We also examined H3K79me2 level in surrounding tissues of nasal processes. H3K79me2 level was not altered in epithelium covering dorsal part of forebrain, neuroepithelium, or in mesenchyme or epithelium of maxillary and mandibular processes (Supplementary Fig. S6d,e,h,i). Together these data demonstrate that Af10 is required during embryogenesis for full H3K79me2 in developing midfacial tissues, including epithelium, mesenchyme and olfactory epithelium of the nasal processes, but this Dot1L cofactor is not needed for H3K79me2 in neuroepithelium.

To further validate that H3K79me2 was diminished in *Mllt10*-KO embryos, we quantified H3K79me2 by western blot analysis. A piece of tissue encompassing the nasal processes, olfactory epithelium, a tiny part of maxillary processes, and small segment of forebrain was dissected out from E10.5 embryos as shown in Fig. 5i. Histones were extracted from the isolated tissue and separated by SDS-PAGE. Histone H3 and H3K79me2 were then detected by western blot. While the histone H3 signal intensity was not altered in mutant tissue, the signal intensity of H3K79me2 was significantly decreased in nasal tissues of *Mllt10*-KO embryos relative to tissues of wild-type embryos (Fig. 5j). Densitometry analysis of each band showed that the level of H3K79me2 in the *Mllt10*-KO tissues was decreased by 35% relative to wild-type embryos (Fig. 5k). Taken together, these data indicate that Af10 functions as a regulator of Dot1L-mediated H3K79me2 in embryonic tissues of the developing face.

### Af10 is required for the expression of the neural crest gene, *AP2α*

**Figure 6:**
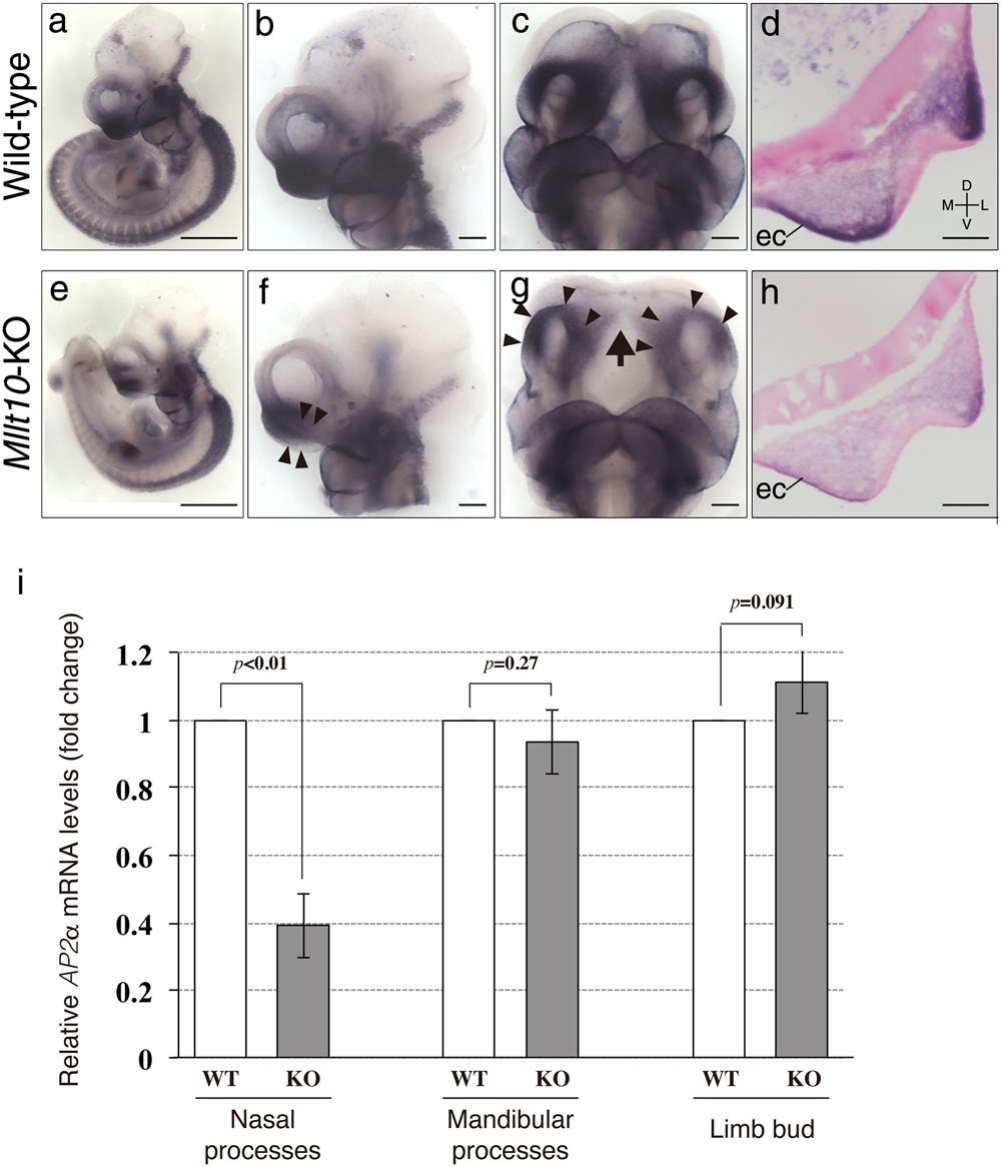
Loss of *Mllt10* causes down-regulation of *AP2α*. Expression of *AP2α* mRNA was detected by whole-mount *in situ* hybridization wild-type embryos (**a**–**d**) and *Mllt10*-KO embryos (**e**–**h**) at E10.5. Lateral views of whole-embryos (**a** and **e**; scale bars 500 μm), lateral views of heads (**b** and **f**; scale bars 100 μm), ventral views of heads (**c** and **g**; scale bars 100 μm), and frontal sections of nasal process (**d** and **h**; scale bars 100 μm) are shown. The decrease of *AP2α* mRNA expression in the nasal processes and the medial region between processes are indicated by arrowheads and arrow, respectively. Over 10 embryos were analyzed. ec; ectoderm. Directional planes for d and h were shown in d (D; dorsal, V; ventral, M; medial and L; lateral). (**i**) Expression level of *AP2α* was quantified by RT-qPCR. Quantities of *AP2α* mRNA were normalized to *Gapdh* mRNA and the relative values are presented as bar graph. WT; wild-type, KO; *Mllt10*-KO. Data are mean ± s.e. of 4 independent experiments. Statistical differences were assessed with Student’s *t*-test, and *p*-values are shown.

Whereas epigenetic methylation of histone H3 at other residues can activate or repress target gene expression, Dot1L-mediated H3K79me functions only to cause target gene activation^13,14^. Thus, the reduction of cell proliferation associated with reduced H3K79me2 in *Mllt10*-KO embryos might be attributed to down-regulation of the expression of gene(s) related to the facial development. Possible targets could include genes associated with pathogenesis of FND or other severe congenital craniofacial anomalies characterized by midline facial cleft^27^. Such genes include those important for proliferation of CNCCs. In order to investigate potential candidate genes that might be targets of Dot1L-mediated H3K79me2 regulation, we utilized whole-mount *in situ* hybridization and RT-qPCR to examine the expression of genes involved in CNCC development, and genes associated with FND pathogenesis in E10.5 embryos. Genes examined included *AP2α*, *Ets1*, *Sox9*, *Msx1*, *Fgf8* and *Shh*, which have been shown to be important for CNCC development^35^, and *Alx1*, *Alx4*, and *Six2* which have been associated with FND^36–38^. From this analysis we identified that *AP2α* was significantly down-regulated in nasal processes of *Mllt10*-KO embryos relative to wild-type embryos (Fig. 6a–h). Lateral and ventral views of embryonic heads showed that *AP2α* expression was decreased in the nasal processes of *Mllt10*-KO embryos (Fig. 6f,g; arrowheads). *AP2α* expression was also reduced in the midline region between the two medial nasal processes (Fig. 6g; arrow). The reduction in *AP2α* expression in *Mllt10*-KO embryos appeared specific for the nasal processes and facial midline, expression was not notably altered in maxillary processes, mandibular processes or pharyngeal arches (Fig. 6e–g). RNA *in situ* hybridization did not reveal obvious changes in expression of other genes screened (Supplementary Fig. S7). The *AP2α* gene is expressed in CNCCs of the nasal processes, and also in the medial ectoderm of the midline. To identify the tissue in which *AP2α* expression was altered within the developing facial region, we sectioned embryos stained for *AP2α* mRNA. Analysis of frontal sections of E10.5 embryos revealed that *AP2α* expression was decreased both in CNCCs, and also in ectodermal cells, of the nasal processes (Fig. 6d,h). These results indicate that *AP2α* expression is reduced in ectodermal and mesenchymal cells of nasal processes of *Mllt10*-KO embryos, demonstrating that Af10 activates *AP2α* expression specifically in nasal processes during embryogenesis.

Having identified that *AP2α* expression was altered specifically within developing facial tissues, we sought to verify that the nasal process-specific reduction in *AP2α* expression was not due to tissue-specific loss of *Mllt10* expression in *Mllt10*-KO embryos. We therefore examined *Mllt10* mRNA levels in different tissues by performing RT-qPCR using cDNA samples prepared from nasal processes, mandibular processes, and forelimb buds of E10.5 embryos. Primers positioned at exon 9 and exon 10, a region not disrupted by the conditional deletion, amplified products in all *Mllt10*-KO samples (Supplementary Fig. S8). The amplification level of the non-disrupted region of *Mllt10* was lower in mutant embryos than of wild-type. Within mutant embryos, there was no significant difference in amplification level between different tissues. A primer set amplifying a segment between exon 16 and exon 17, within the interval deleted by Cre recombination, was used to quantify transcripts from the *Mllt10*-KO-deleted region. No mRNA spanning the *Mllt10*-KO deleted region was detected from any tissues of mutant embryos. Therefore, specific reduction of *AP2α* expression in nasal processes of *Mllt10*-KO embryos was not due to tissue-specific loss of *Mllt10* expression.

RT-qPCR analysis revealed that relative expression level of *AP2α* in nasal processes was decreased to approximately 39% that of wild-type embryos (Fig. 6i). By RT-qPCR, expression of *Six2* could also be detected to be mildly reduced in *Mllt10*-KO mutant nasal processes, being to 80% that of wild type embryos (Supplementary Fig. S9). Owing to the dramatic level of reduction *AP2α* expression, detected both visually and quantitatively, we focused our investigations on *AP2α* as likely a direct target gene of H3K79me.

### H3K79me2 is enriched at the *AP2α* locus in an Af10-dependent manner

**Figure 7:**
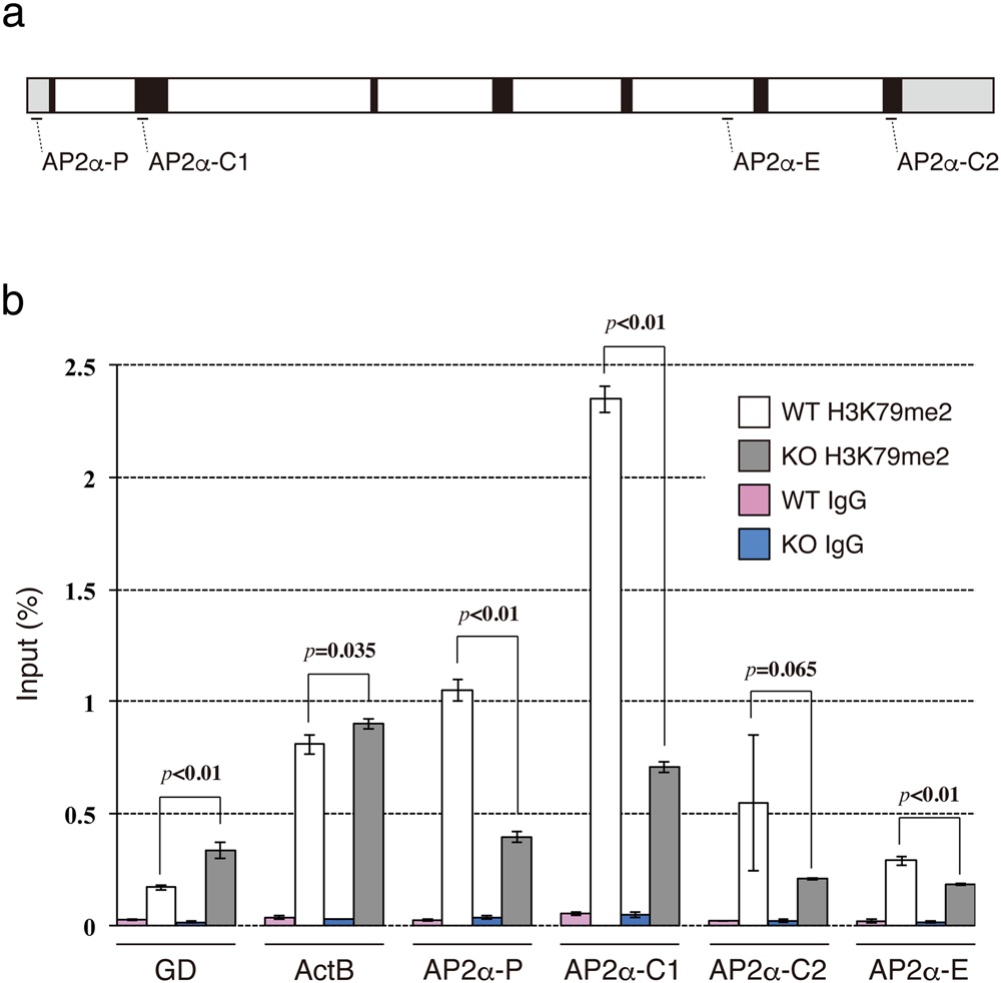
Within nasal processes the *AP2α* promoter, enhancer and coding regions are enriched for Af10-dependent H3K79me2. (**a**) Schematic representation of the mouse *AP2α* locus. The position of qPCR amplicons is indicated. Black box; exon, white box; intron, gray box; untranslated region. (**b**) Chromatin prepared from wild-type (WT) or *Mllt10*-KO (KO) nasal processes was immunoprecipitated using anti-H3K79me2 antibody (H3K79me2) or control rabbit IgG (IgG). Precipitated DNA was subjected into qPCR. Bar graph shows recovery, expressed as percentage of input. Data are obtained from 4 independent experiments. Data are mean ± s.e. Statistical differences were assessed with Student’s *t*-test, and *p*-values are shown. GD; gene desert, ActB; β-actin.

The reduction in *AP2α* in *Mllt10*-KO embryos suggested the possibility that *AP2α* may be a direct target of regulation by Af10-dependent H3K79me2. To examine whether *AP2α* expression is directly activated via Af10-dependent H3K79me2, we performed chromatin immunoprecipitation with an anti-H3K79me2 antibody, followed by qPCR (ChIP-qPCR) for *AP2α* promoter sequences. We designed primers to amplify four segments in or near the mouse *AP2α* gene: “AP2α-P” is a homology region of the human *AP2α* promoter^39^ in the 5′ untranslated region (5′ UTR), “AP2α-C1” and “AP2α-C2” are two segments of *AP2α* coding DNA, and “AP2α-E” is a previously described enhancer element known as craniofacial and limb enhancer DCE^39^ (Fig. 7a). For a negative control segment of DNA expected to be devoid of H3K79me2, primers were designed to amplify a region of gene desert in chromosome 3^40^. For a positive control DNA segment with H3K79me2 unrelated to Af10 function (unpublished data), primers amplifying the highly transcribed gene *β-actin* were chosen because H3K79me2 occurs in highly transcribed genes. ChIP-qPCR analysis showed that H3K79me2 was enriched on all sites in *AP2α* locus of wild-type embryos. Negligible DNA was detected by qPCR amplification following chromatin immunoprecipitation with rabbit IgG, demonstrating the specificity of immunoprecipitation with the anti-H3K79me2 antibody. Compared to wild type embryos, which had robust H3K79me2 at all sites assayed on the *AP2α* locus, in *Mllt10*-KO embryos H3K79me2 marks were significantly reduced at the *AP2α* potential promoter in 5′ UTR, coding sequences in exon 2, and DCE (Fig. 7b). These data strongly suggest that *AP2α* expression is regulated through H3K79me2, and that Af10 directly activates *AP2α* expression through H3K79me2 in nasal processes.

### Suppression of H3K79me can mimic *Mllt10*-KO phenotypes

The observation that *Mllt10*-KO embryos had reduced nasal *AP2α* expression and midline facial cleft in conjunction with diminished H3K79me2 suggested that facial midline cleft defects could be a direct consequence of loss of H3K79me2. In order to determine if reduced H3K79me2 could be the direct cause of midline facial cleft formation, we examined whether suppression of H3K79me2 is sufficient to alter facial morphogenesis via changes in frontonasal cell proliferation and *AP2α* expression. For this purpose, we took advantage of the *ex utero* whole-embryo culture technique^41–43^. The whole-embryo culture enables strict control of timing of treatments and concentration of chemicals to which an embryo is exposed. For suppression of H3K79me2, we used EPZ-5676, a highly selective and low toxicity aminonucleoside inhibitor of Dot1L methyltransferase activity^44^.

**Figure 8:**
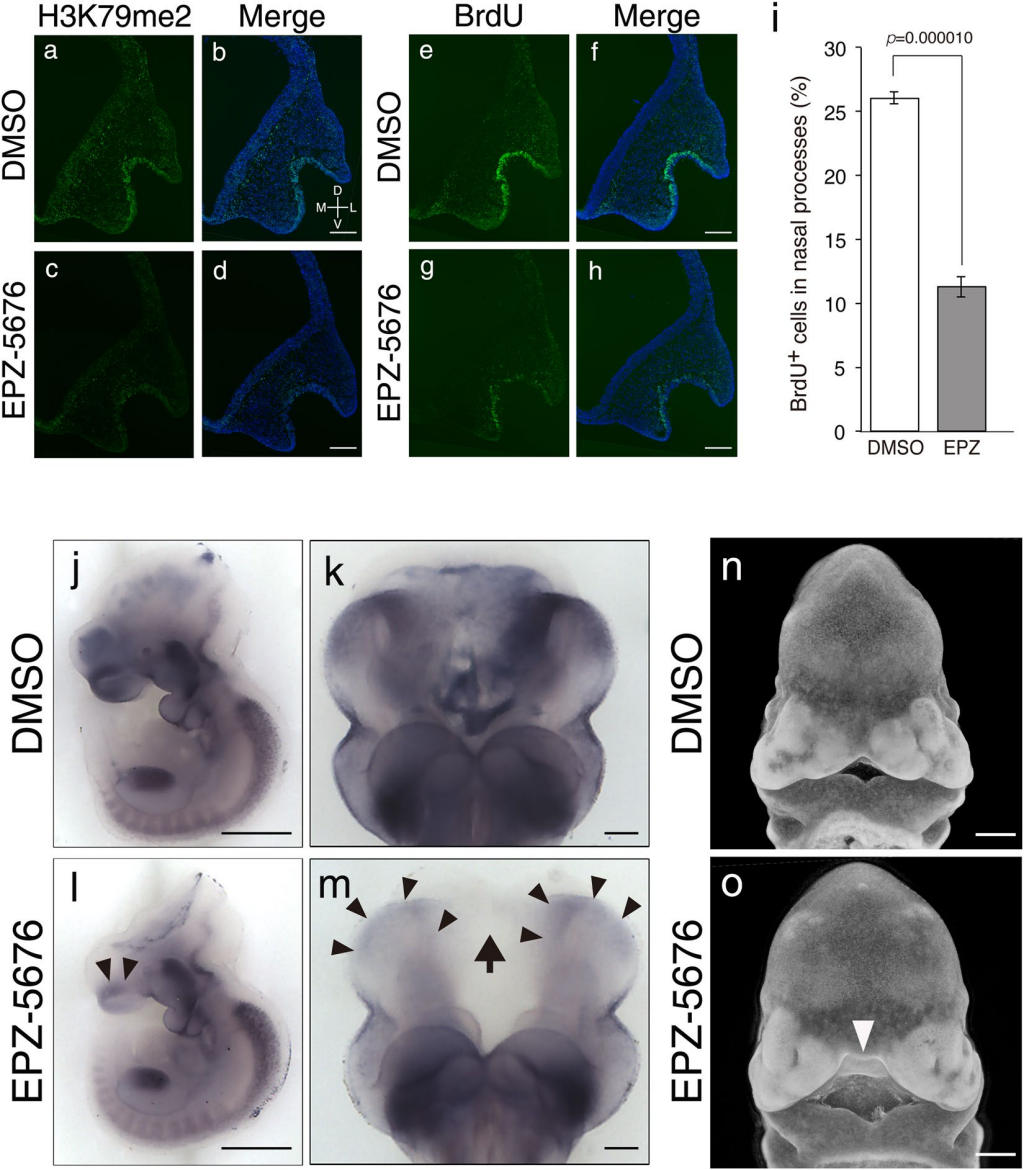
Chemical inhibition of Dot1L in cultured embryos mimics *Mllt10*-KO phenotype. Reduction of H3K79me2 in the nasal process of EPZ-5676-treated embryos (**c** and **d**) relative to DMSO-treated control embryos (**a** and **b**) was confirmed by immunostaining of frontal sections. BrdU-positive proliferating cells in nasal process were detected using frontal sections of DMSO-treated (**e** and **f**) and EPZ-5676-treated (**g** and **h**) embryos. 3–4 histological sections obtained from 3–4 embryos were analyzed. Scale bars; 100 μm. Directional planes for (**a–h**) were shown in (**b**) (D; dorsal, V; ventral, M; medial and L; lateral). (**i**) The number of BrdU-positive cells was counted on 3–4 histological sections obtained from 3 embryos. Data are mean ± s.e. Statistical differences were assessed with Student’s *t*-test, and *p*-values are shown. Expression of *AP2α* mRNA was detected by whole-mount *in situ* hybridization of DMSO-treated embryos (**j** and **k**) and EPZ-5676-treated embryos (**l** and **m**). Lateral views of whole-embryos (**j** and **l**; scale bars 500 μm), and ventral views of heads (**k** and **m**; scale bars 100 μm) are shown. The decrease of *AP2α* mRNA expression in nasal process and its medial region were indicated by arrowheads and arrow, respectively. 5 embryos were analyzed. (**n** and **o**) Craniofacial morphology was analyzed by PseudoSEM. Midline facial cleft induced by EPZ-5676 treatment is indicated by arrowhead. Scale bars; 500 μm. 4 control and 6 EPZ-5676-treated embryos were analyzed.

In order to assay the impact of loss of H3K79me2, E9.5 wild-type mouse embryos were cultured with 10 μM EPZ-5676, or with the vehicle control (DMSO), to the E10.5 stage of development. Following culture, immunostaining was used to assess H3K79me2 in embryos treated with EPZ-5676 or with vehicle. Relative to treatment with vehicle, exposure to EPZ-5676 effectively suppressed H3K79me2 in cultured embryos (Fig. 8a–d). Embryos cultured with EPZ-5676, or vehicle control, were also assessed for cell proliferation and for *AP2α* expression. The percentage of BrdU-positive proliferating cells was significantly reduced in nasal processes of EPZ-5676-treated embryos (Fig. 8g,h,i; 11.3 ± 0.8%) relative to control embryos (Fig. 8e,f,i; 26.0 ± 0.4%), demonstrating that loss of H3K79me2 can cause a reduction in cell proliferation. Notably, the percent of BrdU-positive cells was more strongly reduced following culture treatment with EPZ-5676 relative to control (about 2.3-fold, Fig. 8i), than the reduction observed in *Mllt10*-KO embryos relative to wild type (about 1.3-fold, Fig. 3m; E10.5). *AP2α* expression was also dramatically decreased in nasal processes of EPZ-5676-treated embryos relative to controls (Fig. 8l,m; arrowheads and arrow). The extent of *AP2α* down-regulation following EPZ-5676 chemical inhibition of H3K79me2 appeared to be more severe than that in *Mllt10*-KO embryos (Fig. 6f,g; arrowheads and arrow). As in the mutant model, chemical suppression of H3K79me2 by EPZ-5676 in cultured embryos did not induce ectopic apoptosis in nasal processes (Supplementary Fig. S10). These data demonstrate that chemical inhibition of H3K79me2 can recapitulated the reduction in cell proliferation and down-regulation *AP2α* expression that occurs in *Mllt10*-KO embryos.

Finally, we ascertained whether the chemical suppression of H3K79me2 is able to cause midline facial cleft. We cultured wild-type embryos with or without 10 μM EPZ-5676 from E9.5 until the E11.5 stage of development and analyzed embryo craniofacial morphology. In control cultured embryos, the medial nasal processes were completely fused at the midline (Fig. 8n). In contrast, EPZ-5676-treated embryos had a noticeable gap at the midline between the medial nasal processes (Fig. 8o; arrowhead). These data demonstrate that defects in facial morphology observed *Mllt10*-KO embryos can be phenocopied by suppression of H3K79me2. Collectively, our observations demonstrate that Af10-dependent H3K79me2 is essential for development of nasal processes and adjacent tissue, and subsequent formation of the mid-face.

## Discussion

Here we describe for the first time, a role of *Mllt10* and H3K79me in craniofacial development in mouse embryos. We demonstrated that *Mllt10* is expressed in facial primordia, and that loss of *Mllt10* results in ocular hypertelorism and midline facial cleft. These facial abnormalities in *Mllt10*-KO embryos are reminiscent of FND. FND patients exhibit a spectrum of phenotypes including ocular hypertelorism, wide or cleft nose with abnormal nasal tip, and midline cleft of the philtrum. There are 8 types of FND, and responsible genes have been identified in 6 types so far; FND1, 2, 3 and 4 are caused by the mutation in *ALX3*, *ALX4*, *ALX1* and *SIX2*, respectively. A related syndrome, Craniofrontonasal syndrome (OMIM 304110), which presents in females as FND is caused by mutations of *EFNB1*. Acromelicfrontonasal dysplasia (OMIM 603671) is a distinctive and rare form of FND caused by deficiency of *ZSWIM6* ^36,38,45–49^. For 2 additional types of FND, Oculoauriculofrontonasal syndrome (OAFNS;OMIM 601452) and Acrofrontofacionasal dysplasia (AFFND; OMIM 201180, 239710), the causative gene has not yet been identified^50,51^. Our study suggests that disruption of *Mllt10* could be involved in the pathogenesis of OAFNS and/or AFFND. Clinical studies may elucidate a genetic correlation between those diseases and *Mllt10* in the future. Although 8 familial types of FND have been identified, most cases are thought to be sporadic^52^. Furthermore, it has been reported that phenotypic severity varies even among patients who have same mutation in *ALX3* ^45^. These observations suggest that environmental conditions in the uterus might contribute to the pathogenesis of FND in the embryo, and/or the severity of the phenotype in the offspring. Regulation of gene expression in response to environmental stimuli can be mediated, at least in part, by epigenetic mechanisms. Indeed, the histone methylation status in an embryo can be altered by varying oxygen, nutrition, and intermediate metabolite levels^53,54^. During pregnancy, the intrauterine environment can be impacted by availability of those factors, which influence placental function, or by maternal life style, which determines which environmental factors may reach an embryo. The epigenetic state of an embryo could be influenced by variation in these environmental factors. The impact of environmental factors on midfacial development could be mediated through a mechanism such as Af10-dependent H3K79me2. Disturbances of H3K79me by environmental factors could possibly influence the onset and/or phenotypic severity of FND. In support of this idea, the expression level of *Six2* was reduced by loss of *Mllt10*, suggesting the phenotypic severity of FND4 could be affected by H3K79me state. The discovery that *Mllt10* regulates midfacial development via H3K79me2 is therefore of considerable interest.

Many knockout mice exhibiting midfacial defects have been identified^32^. Among them, knockout of *Srf* ^55^, *Pdgfrα* ^56^, *Alx3/4* ^37^, or *Foxd3* ^57^ exhibit midline facial cleft with accompanying split of nasal septum, phenotypes similar to that observed in *Mllt10*-KO. Despite the phenotypic similarity, the molecular mechanisms causing midfacial developmental defects in these other mutant models are quite different. *Foxd3;Wnt1-cre* and *Alx3/4* double knockout mice exhibit massive apoptosis of CNCCs, resulting in hypoplasia of nasal processes^37,57^. Since *Srf* genetically interacts with signaling by platelet-derived growth factor (PDGF), *Srf* ^*fl/fl*^ *;Wnt1-cre* and *Pdgfrα* ^*fl/fl*^ *;Wnt1-cre* mice exhibit similar phenotypes; hypoplasia of nasal processes caused by proliferation and migration defects of CNCCs^55,56^. Similarly to *Srf* and *Pdgfrα* knockout phenotype, *Mllt10*-KO embryos showed cell proliferaton defects in nasal processes, implying that Af10-dependent H3K79me2 might be involved in the activation of Pdgfrα-SRF pathway. Therefore, it will be important to investigate if the activity of Pdgfrα-SRF pathway and CNCCs migration is compromised by loss of *Mllt10*.

By whole-mount *in situ* hybridization and RT-qPCR analysis we have shown here that *Mllt10* is required for the expression of *AP2α* and *Six2* in the nasal processes. The strong reduction in *AP2α* expression prompted us to investigate the possibility that *AP2α* may be direct target of Af10-dependent H3K79me2. Indeed, by ChIP-qPCR we have demonstrated that Af10-dependent H3K79me2 mark is enriched at the *AP2α* locus. The data are the first to demonstrate a direct regulation of *AP2α* expression by Af10. *AP2α* is one of genes related to neural crest development and *AP2α* knockout embryos exhibit severe midline facial cleft with defects in the neural tube, limb, and body wall. In *AP2α* knockout embryos, at E9.5, premigratory CNCC are formed normally^58,59^, indicating that *AP2α* is required for CNCC development at a later stage, during the process of migration, differentiation, proliferation and/or survival of neural crest cells. Green *et al*. revealed that the cause of midfacial defects in *AP2α* transheterozygotes (Neo/Null) is decreased mesenchymal cell proliferation in the nasal processes^60^. It has also been demonstrated, using chimeric mice composed of wild-type cells and *AP2α*-null cells, that *AP2α* is independently required for distinct developmental processes, suggesting the existence of a mechanism regulating tissue-specific expression of *AP2α* ^61^. Based on these previous findings, and the data we report here, we propose that activation of *AP2α* expression by Af10-dependent H3K79me2 in nasal process is one important mechanism for midfacial morphogenesis. Our data strongly suggest a link between *AP2α* expression and proliferation of CNCC-derived nasal process mesenchyme. The *Mllt10*-KO phenotype cannot be explained by loss of *AP2α* in CNCC-derived mesenchyme alone because neural crest-specific disruption of *AP2α* does not induce midline facial cleft^62^. This discrepancy may suggest that *AP2α* expression in nasal process epithelium and olfactory epithelium, lost in *Mllt10*-KO, may contribute to mesenchymal cell proliferation through non-cell autonomous interactions. Another explanation is the impact of developmental hypertelorism on facial morphogenesis. In *Mllt10*-KO embryos, the forebrain is expanded laterally, which seems to physically influence on midfacial formation. Notably, H3K79me2 level in neuroepithelium of *Mllt10*-KO embryos is comparable with that of wild-type. Thus, Af10 probably regulates forebrain development through a non-cell autonomous mechanism. In future studies it will be important to address how disruption of *Mllt10* causes hypertelorism.

Mutations in *AP2α* have been identified as a responsible gene for Branchiooculofacial syndrome (BOFS; OMIM 113620). BOFS is characterized by midfacial anomalies such as hypertelorism, broad nasal tip and cleft lip/palate^63^. Over 90% cases of BOFS are caused by missense mutations in a hotspot located at exon 4 and 5 of the *AP2α* gene, but there is no specific correlation between genotype and phenotype^64^. Phenotypic variability of BOFS could be affected by differences in levels of *AP2α* expression caused by variation of H3K79me status. Therefore, disturbance of H3K79me state might be a common pathogenic mechanism determining the severity of midfacial defects observed in congenital craniofacial anomalies, such as FND and BOFS. Our findings could contribute to development of therapeutic approaches for prenatal prevention and amelioration of midfacial abnormalities.

Differences in phenotype between *Dot1L* and *Mllt10* knockout embryos indicate that Af10 modulates spatiotemporal activity of Dot1L and consequent H3K79me. Indeed, *Mllt10* is expressed in restricted pattern including facial primordia in E10.5 embryos. However, some tissues expressing *Mllt10* mRNA, such as limbs, maxillary and mandibular processes are not obviously disturbed in *Mllt10*-KO embryos. Loss of *Mllt10* results in the reduced *AP2α* expression in nasal processes, but not in limbs or mandibular processes. One possible explanation for the lack of phenotype in limbs and mandibles would be the existence of alternative Dot1L co-factors in those tissues that regulate spatiotemporal activity of Dot1L there. In support this idea, Dot1L co-factor genes such as *Af4*, *Af9*, and *Af17* show distinct expression patterns and distinct knockout phenotypes^65–67^. Within *Mllt10*-KO embryos H3K79me2 level is not altered in some tissues, such as neuroectoderm, suggesting that different co-factor combinations regulate for tissue-specific H3K79me2.

By ChIP-qPCR we demonstrate here that H3K79me2 level is significantly decreased at the DCE element of *AP2α*, the enhancer specific for limb and craniofacial tissues. The finding explains how nasal process-specific expression of *AP2α* is achieved, though normal limb development in *Mllt10*-KO embryos remains enigmatic. One potential mechanism that may influence tissue-specificity of H3K79me2 function is variant forms histone H3. In addition to enzymatic modification of resident histones, global replacement of existing histones with other variants is thought to contribute to epigenetic regulation^68^. H3K79me has been known to be enriched on the histone variant H3.3, which is detected at transcriptionally active loci in Drosophila and mammals^69,70^. Interestingly, the zebrafish mutant db1092, which has a mutation in histone H3.3, exhibits severe malformations specifically in craniofacial bones derived from CNCCs^71^. It is possible that activation of H3K79me target genes, especially in facial primordia, could be influenced by the pattern and distribution of histone variants.

We have shown here that inhibition of Dot1L activity mimics the *Mllt10*-KO phenotype, demonstrating the direct link between Af10-dependent H3K79me2 and midfacial development. Notably, we observe that EPZ-5676 treatment causes more severe reduction in cell proliferation and *AP2α* expression than does *Mllt10* deficiency. These observed variation is in keeping with the findings of Steger *et al*., which show that conversion of H3K79 monomethylation into di- and trimethylation is associated with a graded enhancement of target gene transcription^72^. Af10 is required for the conversion from mono- into dimethylation of H3K79, but not for di- to trimethylation^26,28^. In contrast, EPZ-5676 suppresses all Dot1L methylation of H3K79, even monomethylation. The increased severity of the phenotype of EPZ-5676-treated embryos, which lack mono-, di-, and tri-methylated H3K79, relative to *Mllt10*-KO embryos, which lack only di-methylated H3K79, suggests that molecule(s) regulating conversion of H3K79 dimethylation into trimethylation may exist and cooperate with Af10 in midfacial development.

## Methods

### Mice

Heterozygous *Mllt10* exon16-deficient (*Mllt10*-Het) mice were maintained on a C57BL/6 background. We intercrossed *Mllt10*-Het mice to obtain homozygous *Mllt10* exon16-deficient (*Mllt10*-KO) mutant embryos. For embryonic staging, the morning of the vaginal plug designated as embryonic day (E) 0.5.

### Generation of *Mllt10* exon16-deficient mice

The *Mllt10* targeting vector was constructed using Diphtheria toxin A (DT-A)-pA/conditional KO FW Plasmid according to protocols provided by RIKEN Center for Life Science Technologies (http://www2.clst.riken.jp/arg/Methods.html). A genomic 555 bp sequence including *Mllt10* exon16 (Targeting site) was amplified by PCR using C57BL/6 Bacterial artificial chromosome library (PR23-349H7, BACPAC) as a template and FW-Af10B and Rev-Af10B primer. The targeting site was inserted into a *Pme*I /*Sac*II restriction site of DT-A-pA/conditional KO FW Plasmid to create *Mllt10* exon16-flox-neo allele. 5′ and 3′ arms (termed Long and Short arm, respectively) were isolated from PR23-349H7 by Red/ET methods (Gene Bridge), and then 7.5-kb Long arm and 2.9-kb Short arm were inserted into *Sal*I/*Not*I and *Xho*I site of DT-A-pA/conditional KO FW Plasmid. The targeting vector was linearized by restriction enzyme digestion with *Sal*I and electroporated into TT2 ES cells^73^. From 140 G418-resistant ES cell colonies, 25 clones were identified as positive for homologous recombination by PCR using Neogt1 and Af10C2 primers. Among the 25 homologous recombined ES cell colonies identified by PCR, 15 colonies were confirmed as having undergone homologous recombination by southern blot analysis. Three clones were injected into respective 8 cell stage embryos. Resulting chimeric males were crossed with C57BL/6 females to achieve germline transmission of *Mllt10* exon16-flox-neo allele in a mixed genetic strain of C57BL/6 and CBA. Heterozygous F1 mice were intercrossed, and subsequently crossed to the Cre deleter strain and FLP deleter strain provided by the RIKEN Center for Life Science Technologies, Animal Resource Development Unit, Japan, to produce *Mllt10* exon16-deficient alleles (Accession No. CDB1033K: http://www2.clst.riken.jp/arg/mutant%20mice%20list.html).

Genotyping of offspring was performed by PCR to amplify genomic DNA specific for the mutant or wild-type allele. To detect wild-type allele, we used primers Af10A and Af10C. To detect mutant allele, we used primers FW-cTV2 and Af10-C. Sequences of primers were as follows; Af10A:5′-CTT-ACAGCTTCGCTATGATCAACCGAGC-3′, FW-Af10B; 5′- GTCGTTTAAACGTTCAACTATTTTACTTTA-3′, Rev-Af10B; 5′- GTCCCGCGGTATGTATTTGTTTGTATTTTG-3′, Neogt1; 5′-CATCGCCTTCTATCGC-CTTCTTGACG-3′, Af10C; 5′-GACAGCCTTTCTATCAAGTTCACAAGACAC-3′, Af10C2; 5′- GCCAGTA-TCATGTGACCGCCACAAAAATGC-3′, FW-cTV2; 5′-GACAGCCTTTCTATCAAGTTCACAAGACAC-3′.

### Southern blot analysis

Genomic DNA was extracted from mouse embryonic fibroblast (MEF) cells, which have *Mllt10* exon 16-deficient allele and Neo casette. Genomic DNA was completely digested with *Avr*II or *Eco*RI, and then 10 μg of digested DNA was loaded onto 0.8% agarose gel. Gel transfer was performed using standard protocol. LP and SP probes were labeled with α ^32^P-dCTP using a Random Primer DNA labeling Kit Ver.2 (Takara). Blotted membranes were hybridized with probes and washed at 65 °C using standard protocol. Hybridized probes were visualized using Typhoon FLA9000 (GE Healthcare).

### Cloning for RNA probe synthesis

A partial coding sequences (2208–2806 from the start codon) of mouse *Mllt10* was amplified by PCR using primers; FW-Af10 SP2; GCCGAATTCGGGGACTCCTGGTGACATTCTAGGAATG and RV-Af10 SP2;  CGCACTAGTT-GAGAGGGGCAGGGTTCTGGGAGATTG. PCR products were cloned into pBlueScript II vector for synthesis of cRNA probe.

### Whole-mount *in situ* hybridization

Whole-mount *in situ* hybridization was performed by standard protocol, which has been described before^74^ with slight modifications. Briefly, embryos were collected and fixed overnight with 4% paraformaldehyde (PFA)/phosphate buffered saline (PBS) at 4°C. Samples were dehydrated in graded methanol (25, 50 and 100%), and then stored at −80^o^C. Samples were rehydrated, and then treated with ProteinaseK for 5–10 minutes at room temperature. Samples were fixed with 4% PFA/PBS, 0.05% glutaraldehyde, 0.1% Tween 20/PBS for 20 min at room temperature. Samples were prehybridized with ULTRAhyb (ThermoFisher) for 1 hour at 68°C. Hybridization was performed using ULTRAhyb containing Digoxygenin-labeled antisense RNA probes for 16 hours at 68°C. After hybridization, samples were washed two times with Washing buffer 1 (50% formamide, 5xSSC[pH 5.5], 1% sodium dodecyl sulfate (SDS)) for 30 min at 68°C, and once with Washing buffer 3 (50% formamide, 2xSSC[pH 5.5], 0.2% SDS) for 1 hour at 68°C. Samples were incubated with 0.5% Blocking Reagent (Roche)/0.1% Tween 20 in Tris buffered saline (TBST) for 2 hours at room temperature and then with anti-DIG-AP antibody (11093274910, Roche; 1/5000) overnight at 4°C. After washing with TBST, Digoxygenin-labeled antisense RNA probes were detected by NBT/BCIP staining.

### Whole-mount nuclear fluorescent imaging

Detailed craniofacial morphology of embryos was analyzed by nuclear fluorescent imaging technique, called “Pseudo SEM” as previously described^75^.

### Immunofluorescence

Mouse tissues were fixed with 4% PFA/PBS for 3 hours at 4°C and cryopreserved in 30% sucrose in PBS. Tissues were embedded in OCT and stored at −80°C until use. Cryostat sections were cut at 10 μm and adhered to glass slides. Sections were washed with PBS, then incubated with 0.1 N HCl for 30 minutes at 37°C. After brief washing with PBS, sections were incubated with 0.5% Triton X-100 in PBS for 15 minutes at room temperature, then incubated with blocking buffer (3% BSA in TBST) for 30 minutes at room temperature. Sections were incubated at 4°C overnight with antibodies to BrdU (347580, BD bioscience; 1/100), H3K79me2 (ab3594, Abcam; 1/2000), Histone H3 (sc-10809, Santa Cruz; 1/2000), phosphorylated histone H3 (06–570, Millipore; 1/500) and cleaved caspase-3 (9661, Cell Signaling; 1/200). Sections were washed three times with TBST for 10 minutes and then incubated with appropriate secondary antibodies conjugated with Alexa 488 or 546 (A11001, A21208, Invitrogen; 1/300) for 1 hour at room temperature. Nuclei were stained with DAPI. Fluorescence microscopy was performed on a BX51 microscope with DP30BW CCD camera (Olympus) using 10x and 20x objective lenses. Images were collected with DP controller software (Olympus).

### Preparation of total histone proteins

Tissues including nasal processes, small part of maxillary processes and forebrain were dissected out from E10.5 wild-type and *Mllt10*-KO embryos as shown in Fig. 5i were dissected from E10.5 wild-type and *Mllt10*-KO embryos. Total histone proteins were isolated using EpiQuick Total Histone Extraction Kit (Epigentek) according to the manufacturer’s instructions. The amount of histones was quantified using Pierce BCA Protein Assay kit (Thermo Scientific).

### Western blot analysis

Af10 proteins (full length and a C-terminal truncated form) were analyzed by SDS-PAGE followed by western blotting using anti-Af10/Mllt10 antibody (HPA005747, Sigma; 1/500). β-actin was detected as an internal control using Anti- β-actin antibody (A5441, Sigma; 1/1000).

H3K79me2 levels were analyzed by SDS-PAGE followed by western blotting using anti-H3K79me2 antibody (ab3594, Abcam; 1/3000). Histone H3 was detected as an internal control using Anti-Histone H3 antibody (ab4558, Abcam; 1/9000). We analyzed each band by densitometry with ImageJ software, and normalized against Histone H3 value to quantify levels of H3K79me2 in wild-type and *Mllt10*-KO embryos.

### RT-qPCR

Tissues including nasal processes, small part of maxillary processes and forebrain (Fig. 5i), mandibular processes and forelimb buds were dissected out from E10.5 wild-type or *Mllt10*-KO embryos. Total RNA was extracted from nasal processes using ISOGEN II (Nippon gene) according to the manufacturer’s instructions, and then reverse transcribed using ReverTra Ace -α- (Toyobo) according to the manufacturer’s instructions. cDNA was synthesized from mandibular processes or forelimb buds using SuperPrep Cell Lysis & RT Kit for qPCR (TOYOBO) according to the manufacturer’s instructions. RT-qPCR with SYBR green detection was performed using StepOne Real-Time PCR System (Thermo Fisher). Gene expression was normalized to that of *Gapdh* transcripts. The specificity of PCR reactions was confirmed by no-primers controls at each run. The following primers were used; *AP2α* exon3-4 FW; CCACTCCTTACCTCACGCCA, *AP2α* exon3-4 RV; CACCGAAGAGGTTGTCCTTGT, *Af10* cording FW; AGGAAGTCTCTGCCCATACCT, *Af10* cording RV; CCCTTTGACCTGAGCTGTGA, *Af10* SP FW; AGGGGACTCCTGGTGACAT, *Af10* SP RV;  AGACGTTCTTTCTTGGCAGT, *Ets1* FW; TCGATCTCAAGCCGACTCTC, *Ets1* RV; GATTCCCAGT-CGCTGCTGT, *Shh* FW; GTGGAAGCAGGTTTCGACTG, *Shh* RV; ACGTAAGTCCTTCACCAGCTT, *Msx1* FW; AGAAGATGCTCTGGTGAAGGC, *Msx1* RV; TGTGGTGAAAGGCGTCCTG, *Alx4* FW; CCTGCTA-CGCCAAAGAGAGC, *Alx4* RV; CCCTGTCTCCTTCACACTGAG, *Alx1* FW; ATGGAGACGC-TGGACAATGAG, *Alx1* RV; GCTCTATTCAGCTCGGTGTGA, *Six2* FW; AGGCCAAGGAAAGGGAGAACA, *Six2* RV; GAACTGCCTAGCACCGACTT, *Fgf8* FW; TTGGAAGCAGAGTCCGAGTT, *Fgf8* RV;GTGAATA-CGCAGTCCTTGCC, *Sox9* FW;﻿ GTACCCGCATCTGCACAAC, *Sox9* RV;﻿ CTCCTCCACGAA-GGGTCTCT,*Gapdh* FW;  CATGTTCCAGTATGACTCCACTC, *Gapdh* RV; GGCCTCACCCCATTTGATGT.

### Chromatin Immunoprecipitation (ChIP) assay

Tissue including nasal processes (as shown in Fig. 5i) was incubated with 0.25% Trypsin for 5–6 minutes at 37 °C. Trypsin digestion was quenched by an addition of Dulbecco’s Modified Eagle’s Medium (DMEM) containing 10% FBS. Cells were collected by centrifugation (2000 rpm, 5 minutes), and then resuspended in RPMI-1640 medium containing 10% FBS. Cells were fixed with 1% formaldehyde in RPMI-1640 medium containing 10% FBS for 7 minutes at room temperature, and subsequently quenched by addition of 125 mM glycine to the cell suspension, followed by incubation for 10 min on ice. The formaldehyde-fixed cells were lysed in appropriate volume (1~2 × 10^7^ cells/ml) of FA lysis buffer (50 mM Hepes-NaOH (pH 7.5), 140 mM NaCl, 1 mM EDTA pH 8.0, 1% Triton X-100, 0.1% DOC and 0.1% SDS). Cell lysates were sonicated to shear chromatin to an average length of <0.5 kb by the S220 Focused-ultrasonicator (Covaris), and then fragmented chromatins were collected by centrifugation (15000 rpm, 30 minutes). The chromatin samples were diluted 1/10 with FA dilution buffer (1% triton X-100, 2 mM EDTA, 150 mM NaCl and 20 mM Tris-HCl (pH8.0)). Anti-H3K79me2 antibody (2 μg, ab3594, Abcam) was added into samples and incubated on a rotating wheel (overnight, 4 °C). 20 μl of Dynabeads protein G (Invitrogen) per 2 μg of antibody was added into sample, followed by incubation for 4–6 h at 4 °C. Dynabeads protein G-antibody complex was washed with FA wash buffer (0.1% SDS, 1% TritonX-100, 2 mM EDTA, 20 mM Tris-HCl pH 8.0 and 150 mM NaCl) 4 times, and then washed with PBS containing 0.5% TritonX-100 twice. Dynabeads protein G-antibody complex was suspended in 250 μl elution buffer (1% SDS and 0.1 M NaHCO_3_), and incubated for 15 minutes at room temperature to elute chromatin. Elution was repeated twice. Cross-linking of immunoprecipitated comlex was reversed by an addition of 20 μl of 5 M NaCl and incubation at 65°C. 10 μl of 0.5 M EDTA, 20 μl of Tris-HCl (pH 6.5) and 2 μl of 20 mg/ml proteinase K (Wako) were added, followed by incubation for 2 hours at 55°C. DNA was extracted by phenol/chloroform extraction and collected by isopropanol precitipation with Etachinmate (Wako). Immunoprecitipated DNA and input DNA were analyzed by qPCR, using Applied Biosystems 7500 Fast Real-Time PCR System and FastStart Universal SYBR Green Master (Rox) (Roche). Following primers were used; For negative control (Gene desert on Chromosome 3^40^), Chr.3 desert FW;  ATAGGTACACCAAGGACAGTTAGGA and Chr.3 desert RV; AGTTATCACATTTTCAGAGCCCA. For positive control (β-actin coding region), ActB coding FW; TCCTGGCCTCACTGTCCAC and ActB coding RV; GTCCGCCTAGAAGCACTTGC. For *AP2α* promoter (homology region of human *AP2α* promoter^39^ in mouse *AP2α* exon 1, −291 to −222 from ATG start codon), 0.2up FW; AGGATAGAGATCGTGGGTTCGA and 0.2up RV; GGATCCATCCGAACTTGTACCA. For 5′ side of *AP2α* coding region (sequences in mouse *AP2α* exon 2, 181 to 244 from ATG start codon), Coding-1 FW; CCCCAGTCGCAAGATCCTTA and Coding-1 RV; GGGCGTGCAGAGGATTCA. For 3′ side of *AP2α* coding region (sequences in mouse *AP2α* exon 7, 1068 to 1124 from ATG start codon), Coding-2 FW; GCGGCCCAATCCTATCCT and Coding-2 RV; AGGTTGAAGTGGGTCAAGCAA. For *AP2α* craniofacial enhancer DCE^39^ (sequences in mouse *AP2α* intron 5), DCE-1 FW; GATCCGCACCATTTTATGGAA and DCE-1 RV; TGCGCCAATTAGAGCATCAA.

### Whole-embryo *ex utero* culture

Whole-embryo culture was performed as previously described^41–43^. Briefly E9.5 embryos were dissected and cultured in 100% rat serum supplemented with 2% glucose in the presence of 10 μM EPZ-5676 (Sigma) or 0.1% DMSO as control. For labeling of proliferating cells, BrdU (25 μg/ml) was added at 24 hours after (E10.5) and embryos were cultured further for 1 hour. Embryos were fixed with 4% PFA/PBS for 3 hours at 4°C for immunofluorescence, for 16 hours at 4°C for whole-mount *in situ* hybridization. For whole-mount nuclear fluorescent imaging, embryos were cultured from E9.5 to E11.5, and then fixed with 4% PFA/PBS for 16 hours at 4°C.

### Statistics

For statistical analysis, 2-tailed Student’s *t*-tests were used to determine *p*-values. *p*-values of less than 0.01 were considered significant.

### Study approval

All experiments in this study were carried out in accordance with the regulations and guidelines of Nara Women’s University and Doshisha University. Mice were housed in the animal facility in Department of Biological Science, Nara Women’s University. Welfare guidelines and procedures were performed with approval of Nara Women’s University, Doshisha University animal committee, and Institutional Animal Care and Use Committee of RIKEN Kobe Branch.

### Data Availability

All data generated or analyzed during this study are including in this published article and its Supplementary Information files.

## Electronic supplementary material

Supplementary information
