# Supplementary material for: Mllt10 knockout mouse model reveals critical role of Af10-dependent H3K79 methylation in midfacial development
Source: Sci Rep. 2017 Sep 20;7:11922. doi: 10.1038/s41598-017-11745-5 (PMC5607342; doi:10.1038/s41598-017-11745-5)

Supplementary Information

Mllt10 knockout mouse model reveals critical role of Af10-dependent H3K79 methylation in midfacial development

Honami Ogoh[1], Kazutsune Yamagata[2], Tomomi Nakao[1], Lisa L. Sandell[3], Ayaka Yamamoto[1], Aiko Yamashita[1], Naomi Tanga[1], Mai Suzuki[1], Takaya Abe[4], Issay Kitabayashi[2], Toshio Watanabe[1,*] and Daisuke Sakai[5,*]

[1]Department of Biological Science, Graduate School of Humanities and Science, Nara Women's University, Nara, Japan.
[2]Division of Hematological Malignancy, National Cancer Center Research Institute, Tokyo, Japan.
[3]Department of Oral Immunology and Infectious Diseases, University of Louisville, School of Dentistry, Louisville, KY, USA.
[4]Animal Resource Development Unit and Genetic Engineering Team, RIKEN Center for Life Science Technologies, Kobe, Japan.
[5]Laboratory of Developmental Neurobiology, Graduate School of Brain Science, Doshisha University, Kyoto, Japan.

*To whom correspondence should be addressed at:
Corresponding author; Daisuke Sakai, Laboratory of Developmental Neurobiology, Graduate School of Brain Science, Doshisha University, 1-3 Tatara-Miyakodani, Kyotanabe, Kyoto, 610-0394, Japan. E-mail: dsakai@mail.doshisha.ac.jp
Co-corresponding author; Toshio Watanabe, Department of Biological Science, Graduate School of Humanities and Science, Nara Women's University, Kita-uoya Nishi-machi, Nara, Nara 630-8506, Japan. E-mail: toshiwatana@cc.nara-wu.jp.

Legends to Supplementary Figures

Supplementary FigureS1. Generation of *Mllt10* conditional allele, and phenotype of knockout embryos. (a) Schematic representation of wild-type genomic locus of *Mllt10* with 8 exons (black boxes), introns (lines) and restriction enzyme sites of *Avr*II (A) and *Eco*RI (E) are shown. (b) Targeting vector containing 2 *loxP* sites (black arrowheads) and 2 *frt* sites (white arrowheads), a PGK-Neo-polyA cassette for positive selection, and a MC1-DT-A-polyA cassette for negative selection is represented. (c) The targeted $Mllt10^{flox}$ allele after homologous recombination is shown. (d) The exon16-deleted allele following Cre recombination is shown. (e) The exon16-deleted allele after deleting the PGK-Neo-polyA cassette by Flp recombination is shown. (f, g) Southern blot analysis of genomic DNA from MEF cells wild-type, heterozygous, or homozygous for the conditional floxed allele depicted in (d), hybridized with the LP and SP probe depicted in (a) are shown. (f) Southern blot of *Avr*II digested genomic DNA hybridized with the LP probe. (g) Southern blot of *Eco*RI digested genomic DNA hybridized with the SP probe. WT; wild-type, Het; *Mllt10*-Het, KO; *Mllt10*-KO. The original image is presented in Supplementary Information. (h) PCR genotyping results from wild-type (WT), heterozygous (Het) and homozygous (KO) *Mllt10* exon16-deleted mutants are shown. ΦX174/*Hae*III was used as DNA marker. Primers used for genotyping are indicated at left side of the panels. The original image is presented in Supplementary Information. (i) Western blot analysis for detection of full-length Af10 protein (approximately 110kDa) and C-terminal truncated Af10 protein (approximately 70kDa) is shown. β-actin was detected as an internal control. Full-length protein is completely absent in MEF cells derived from *Mllt10*-KO (KO). WT; wild-type, Het; *Mllt10*-Het, KO; *Mllt10*-KO. The original image is presented in Supplementary Information. (j) *Mllt10*-KO embryos from *Mllt10*-Het intercrosses arrested the development before E16.5 (dead). (k) At E13.5, *Mllt10*-KO embryos are alive, but exhibit swelling of the body due to severe hemorrhage and edema (KO). Scale bars; 1 mm.

Supplementary FigureS2. *Mllt10*-KO embryos have no obvious phenotype of mandible. (a, b) Lateral view of wild-type and *Mllt10*-KO embryos at E12.5. (c, d) Oral view of mandible of wild-type and *Mllt10*-KO embryos. (e, f) Ventral view of mandible

of wild-type and *Mllt10*-KO embryos. md; mandible, t; tongue. Scale bars; 500 μm. 3 embryos were analyzed.

Supplementary FigureS3. *Mllt10*-KO embryos have no obvious phenotype of limbs. (a, b) Dorsal view of forelimb and hindlimb of wild-type and *Mllt10*-KO embryos at E12.5. fl; forelimb, hl; hindlimb. Scale bars; 500 μm. Expression of *Sox9* mRNA was detected by whole-mount *in situ* hybridization of forelimb (c, d) and hindlimb (e, f) of wild-type or *Mllt10*-KO embryos. Chondrogenic mesenchymal condensations appear normal in *Mllt10*-KO embryos. Scale bars; 500 μm. 3 embryos were analyzed.

Supplementary FigureS4. Mesenchymal cells in *Mllt10*-KO nasal processes exhibit mitotic defects. pH3-positive mitotic cells in nasal processes were detected in frontal sections of wild-type (a-c) and *Mllt10*-KO (d-f) embryo heads at indicated embryonic stages. 2 histological sections obtained from 3-4 embryos were analyzed. Scale bars; 100 μm. Directional planes for a-f were shown in (a) (D; dorsal, V; ventral, M; medial and L; lateral).

Supplementary FigureS5. Apoptosis is not induced in nasal process of E10.5 *Mllt10*-KO embryos. Apoptotic cells in nasal process were detected in frontal sections of wild-type (a-c) and *Mllt10*-KO (d-f) embryo heads at indicated embryonic stages using anti-cleaved Caspase 3 antibody. 2 histological sections obtained from 3 embryos were analyzed. Scale bars; 100 μm. Directional planes for a-f were shown in (a) (D; dorsal, V; ventral, M; medial and L; lateral).

Supplementary FigureS6. Proliferation and H3K79me2 levels were not altered in tissues surrounding nasal process mesenchyme in *Mllt10*-KO. (a) Schematic illustration of the regions surrounding nasal process mesenchyme for which proliferation was assessed, and histogram of pH3-positive cell counts within those regions. Bar graph depicts the percentage of pH3-positive cells relative to the total cell number in each tissue of E10.5 wild-type (WT) and *Mllt10*-KO (KO) embryos. Ol epi.; olfactory epithelium, MN epi.; medial nasal epithelium, LN epi.; lateral nasal epithelium, Neuroepi.; neuroepithelium, Mn mes.; mandibular mesenchyme, Mn epi.; mandibular epithelium. The number of pH3-positive cells was counted on 3 histological sections

obtained from 3 different embryos. Data are mean ± s.e. Statistical differences were assessed with Student's *t*-test, and *p*-values are shown. H3K79me2 was visualized by immunostaining using frontal sections at nasal process level (b, c, f, g) and mandibular level (d, e, h, i) of wild-type and *Mllt10*-KO embryos. 3 histological sections obtained from 3-4 embryos were analyzed. Scale bars; 100 μm. Directional planes for d, e, h and i were shown in (e) (D; dorsal, V; ventral, M; medial and L; lateral).

Supplementary FigureS7. RNA *in situ* hybridization screen for altered expression of facial midline morphogenesis genes in *Mllt10*-KO embryos. Expression of mRNA was detected by whole-mount *in situ* hybridization of E10.5 wild-type embryos and *Mllt10*-KO embryos. Lateral and ventral views of heads are shown. Scale bars; 200 μm. 3-6 embryos were analyzed to each gene.

Supplementary FigureS8. Levels of *Mllt10* mRNA from coding region and deleted exon16 region are equivalent in nasal processes, mandibular processes, and limb buds of *Mllt10*-KO embryos. Expression level of *Mllt10* was quantified by RT-qPCR using 2 primer pairs; "coding" amplifying exon 9-10, and "ex.16" amplifying exon 16-17, the region deleted by Cre recombination. Quantities of *Mllt10* mRNA were normalized to *Gapdh* mRNA and the relative values are presented as bar graph. Data are mean ± s.e. of 4 independent experiments. Statistical differences were assessed with Student's *t*-test, and *p*-value are shown.

Supplementary FigureS9. Expression level of potential *Mllt10* target genes in nasal processes assessed by RT-qPCR. Quantities of mRNA were normalized to *Gapdh* mRNA and the relative values are presented as bar graph. Data are mean ± s.e. of 3-4 independent experiments. Statistical differences were assessed with Student's *t*-test, and *p*-values are shown.

Supplementary FigureS10. Apoptosis is not induced by EPZ-5676 treatment. Apoptotic cells in nasal process were detected in frontal sections of DMSO-treated control (a, b) and EPZ-5676-treated (c, d) embryo heads using anti-cleaved Caspase 3 antibody. 3-4 histological sections obtained from 2 embryos were analyzed. Scale bars; 100 μm. Directional planes for a-d were shown in (b) (D; dorsal, V; ventral, M; medial

and L; lateral).

Supplementary Figure S1

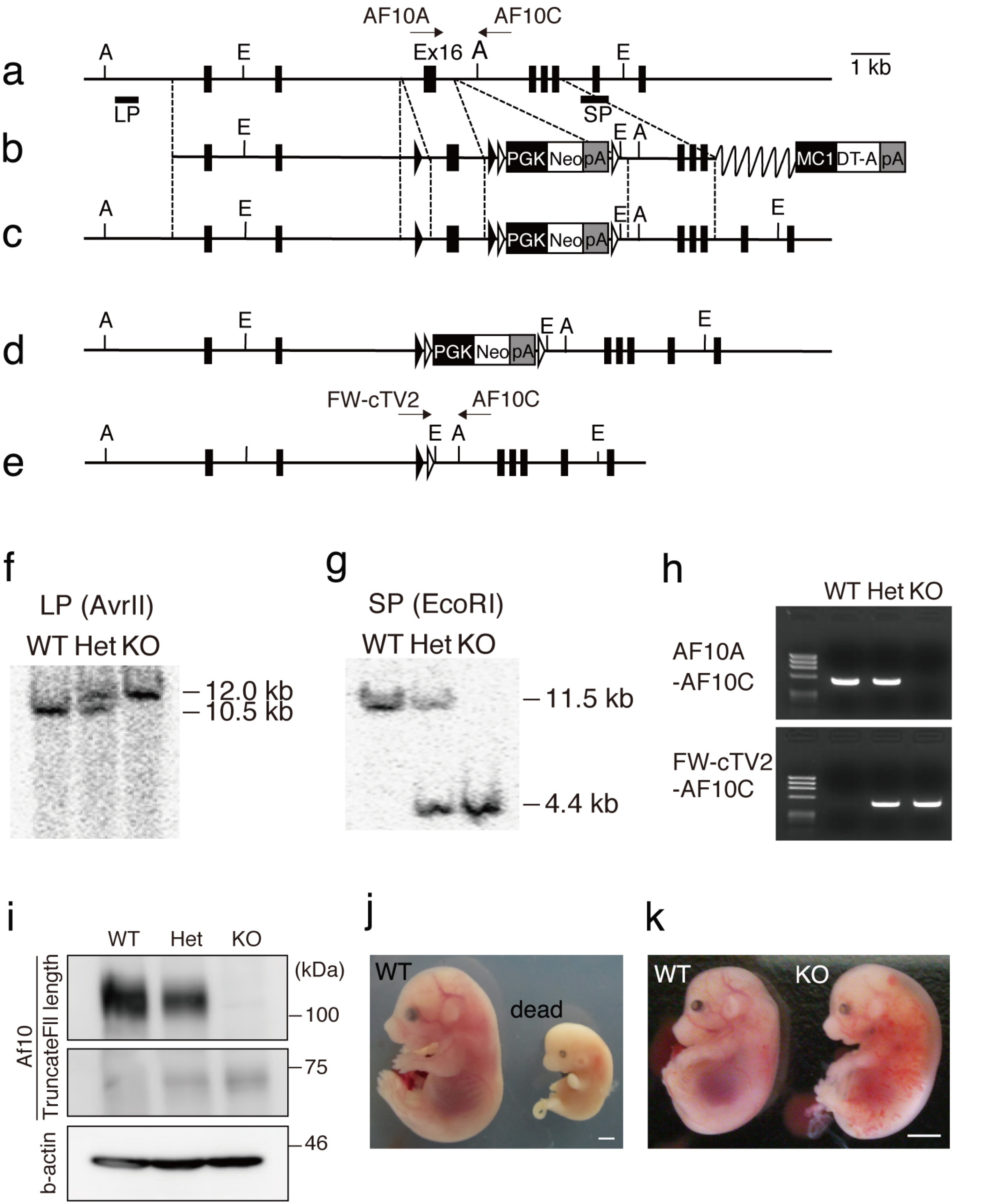

Supplementary Figure S2

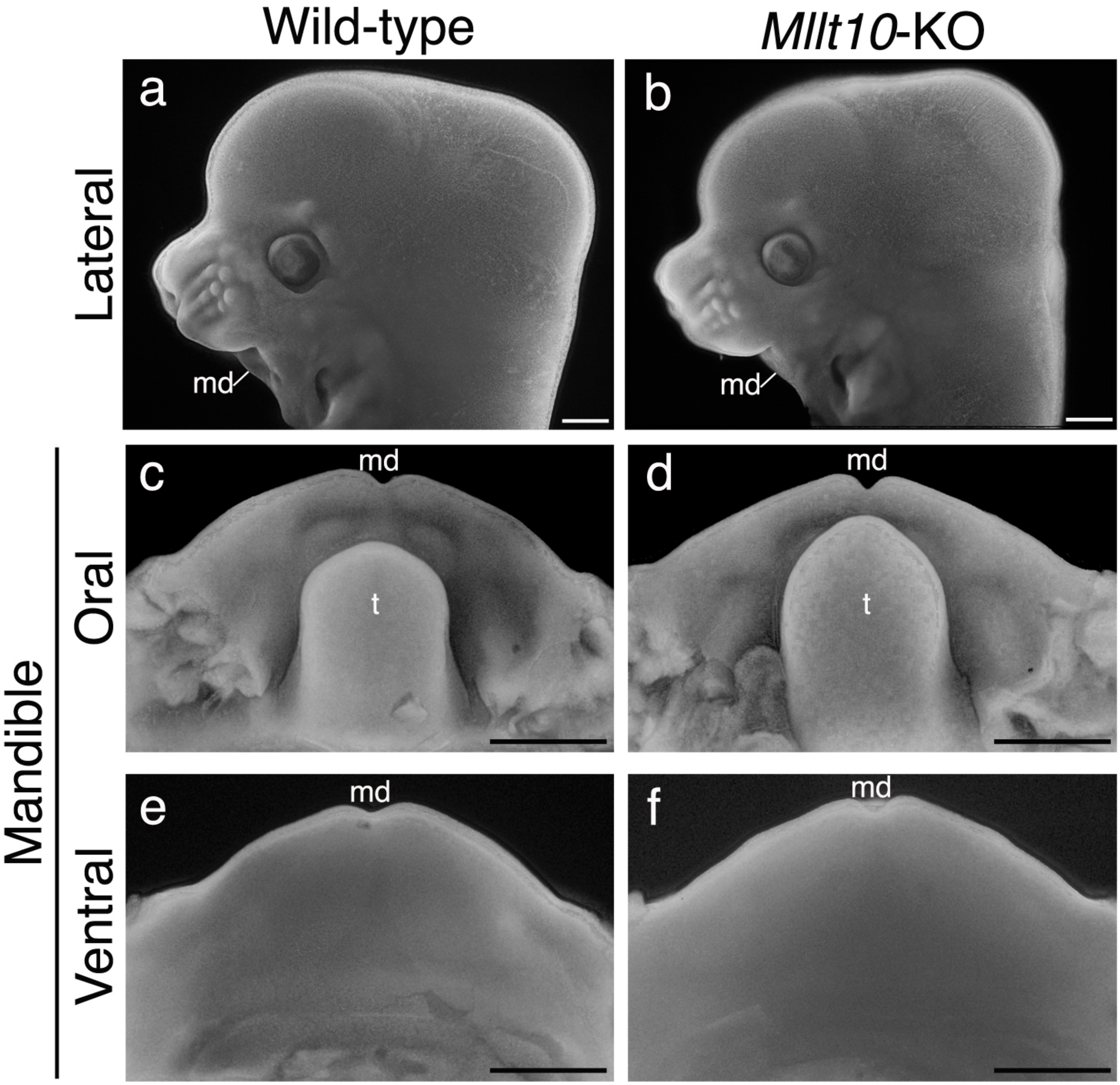

Supplementary Figure S3

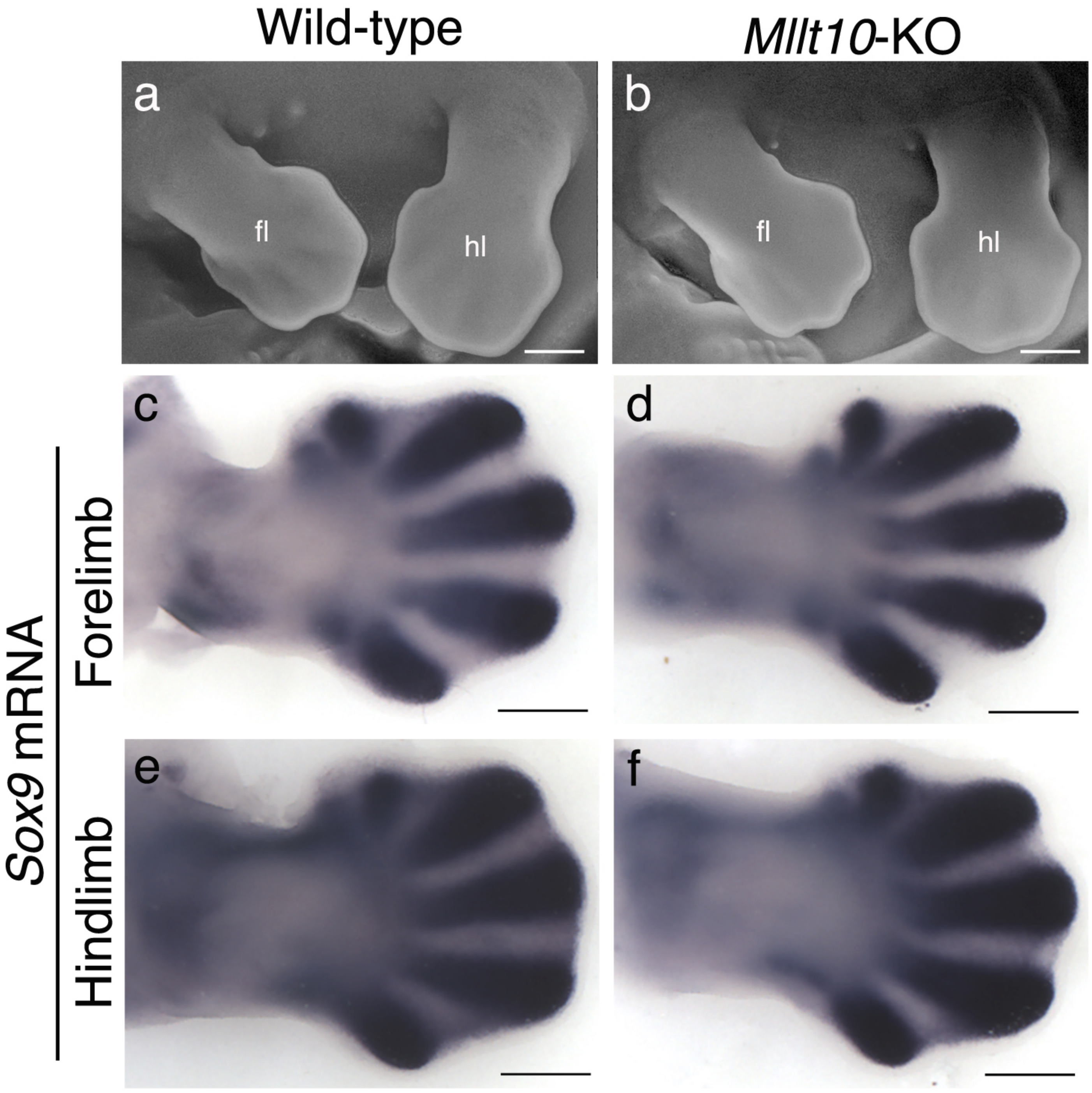

Supplementary Figure S4

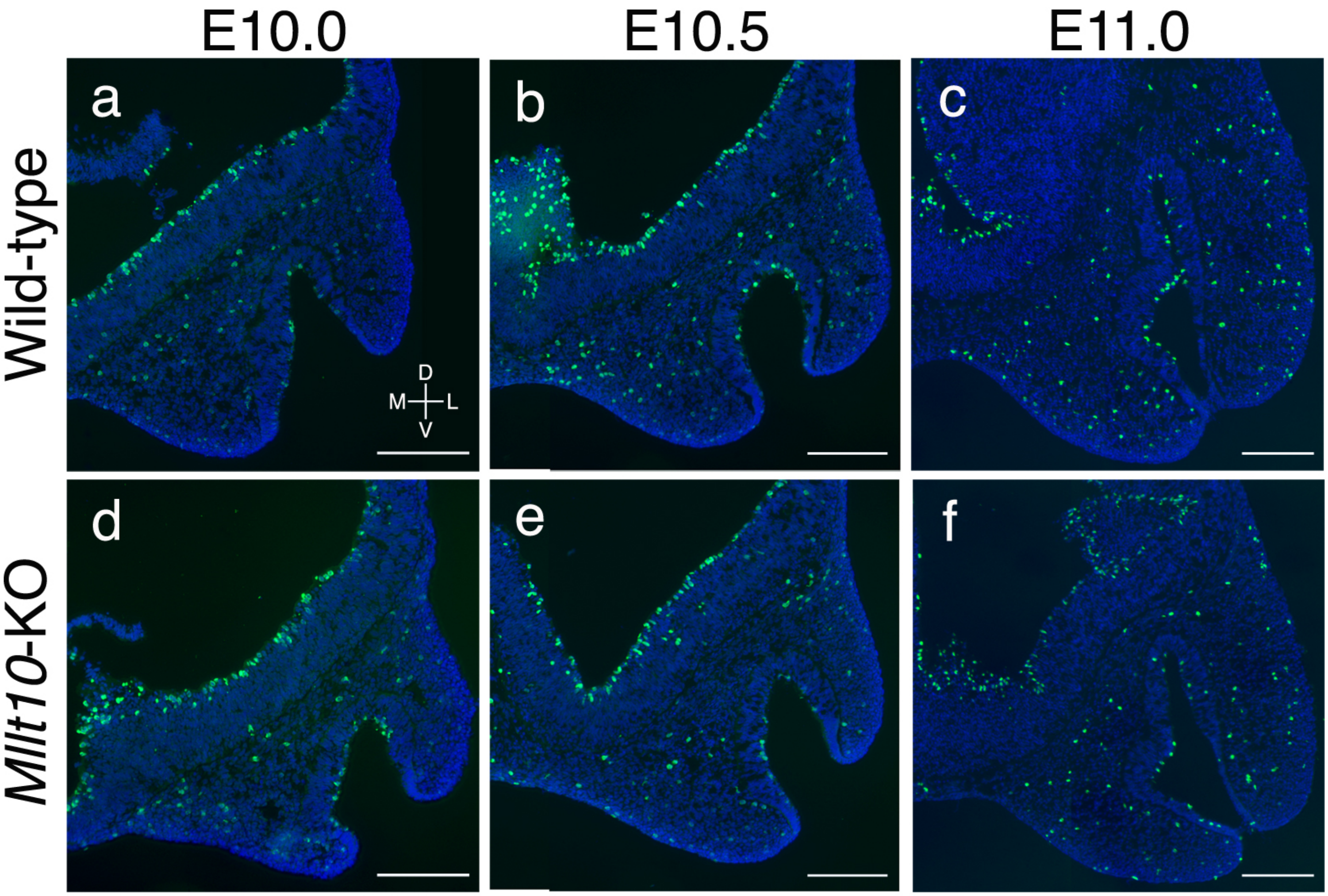

Supplementary Figure S5

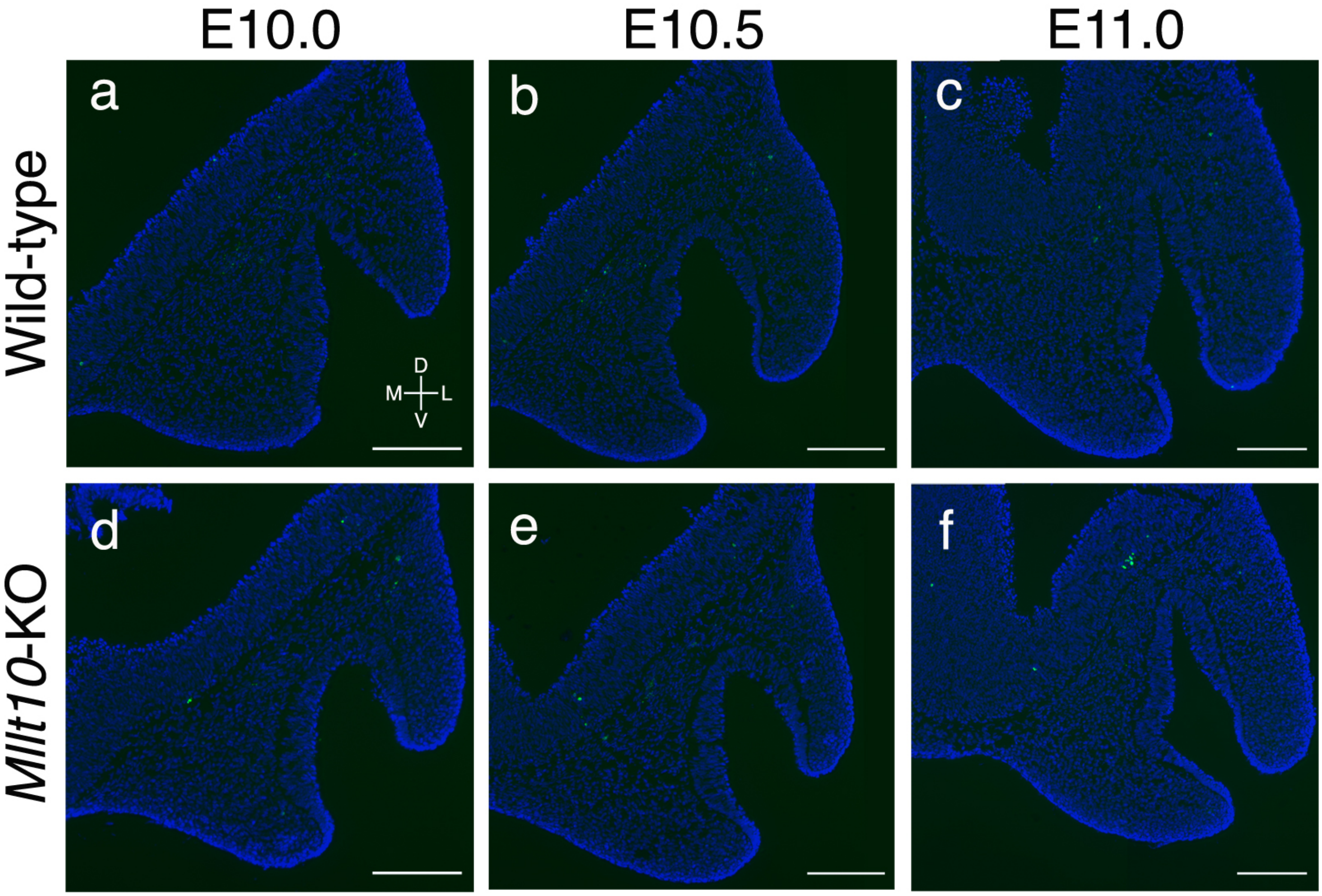

Supplementary Figure S6

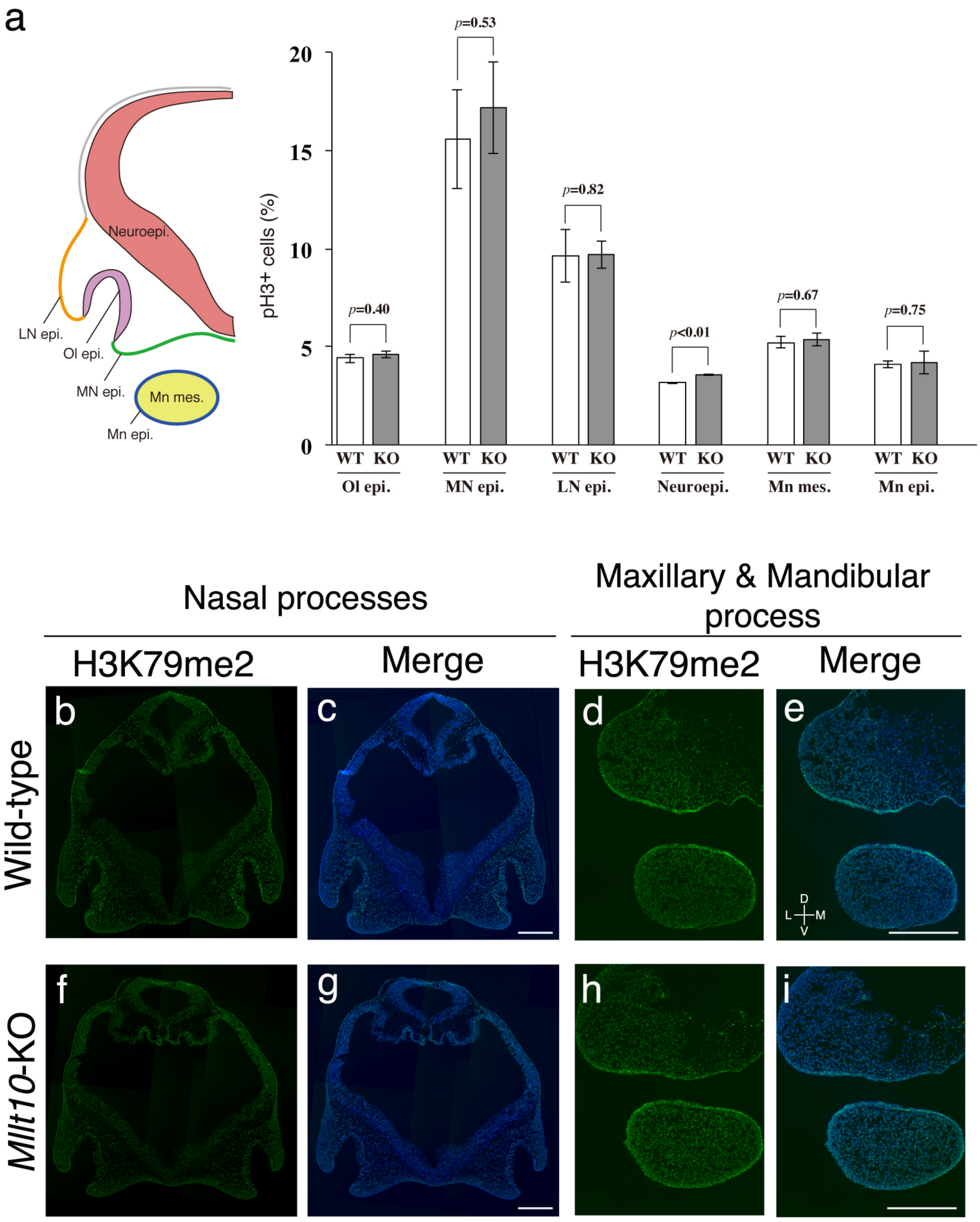

Supplementary Figure S7

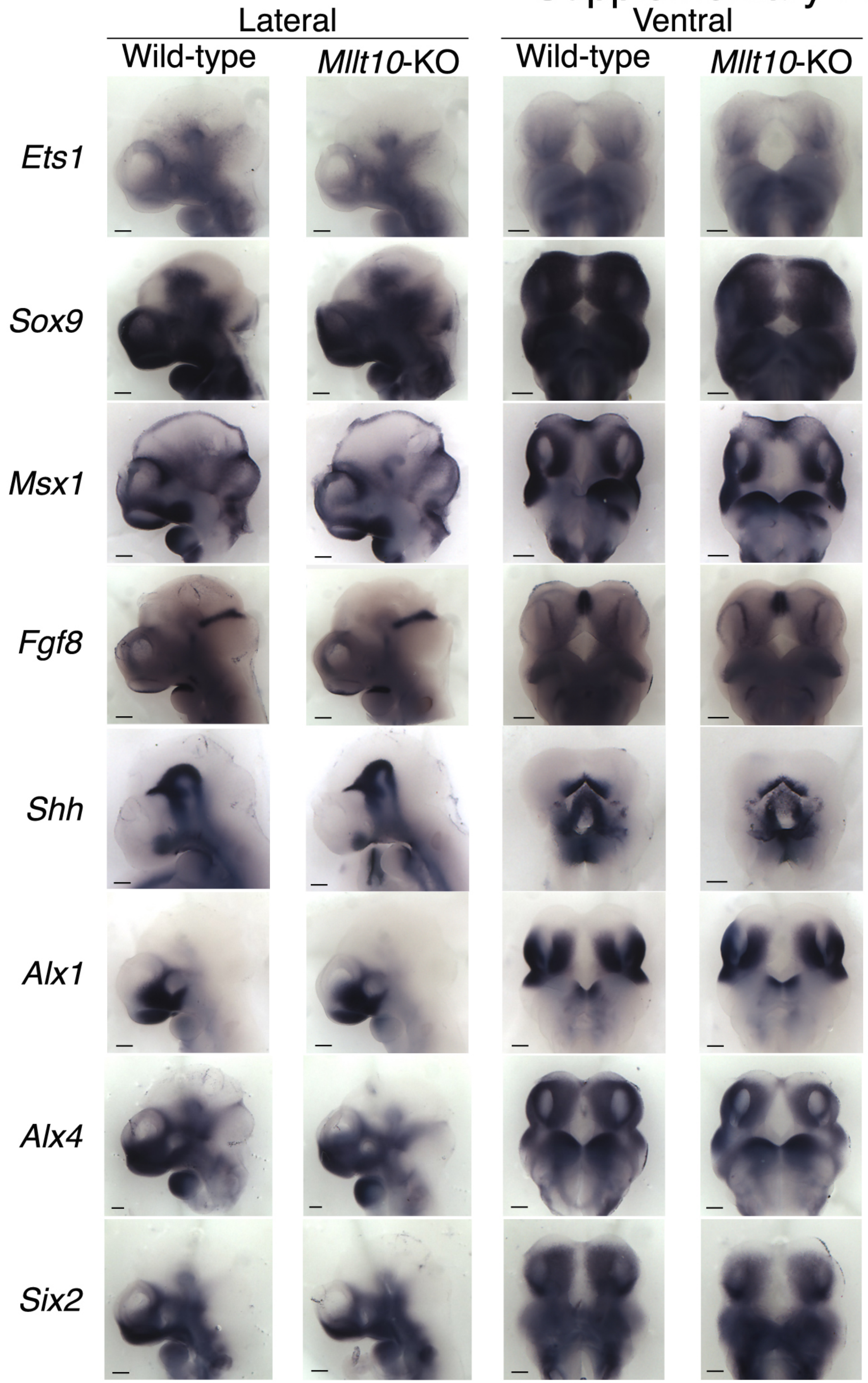

Supplementary Figure S8

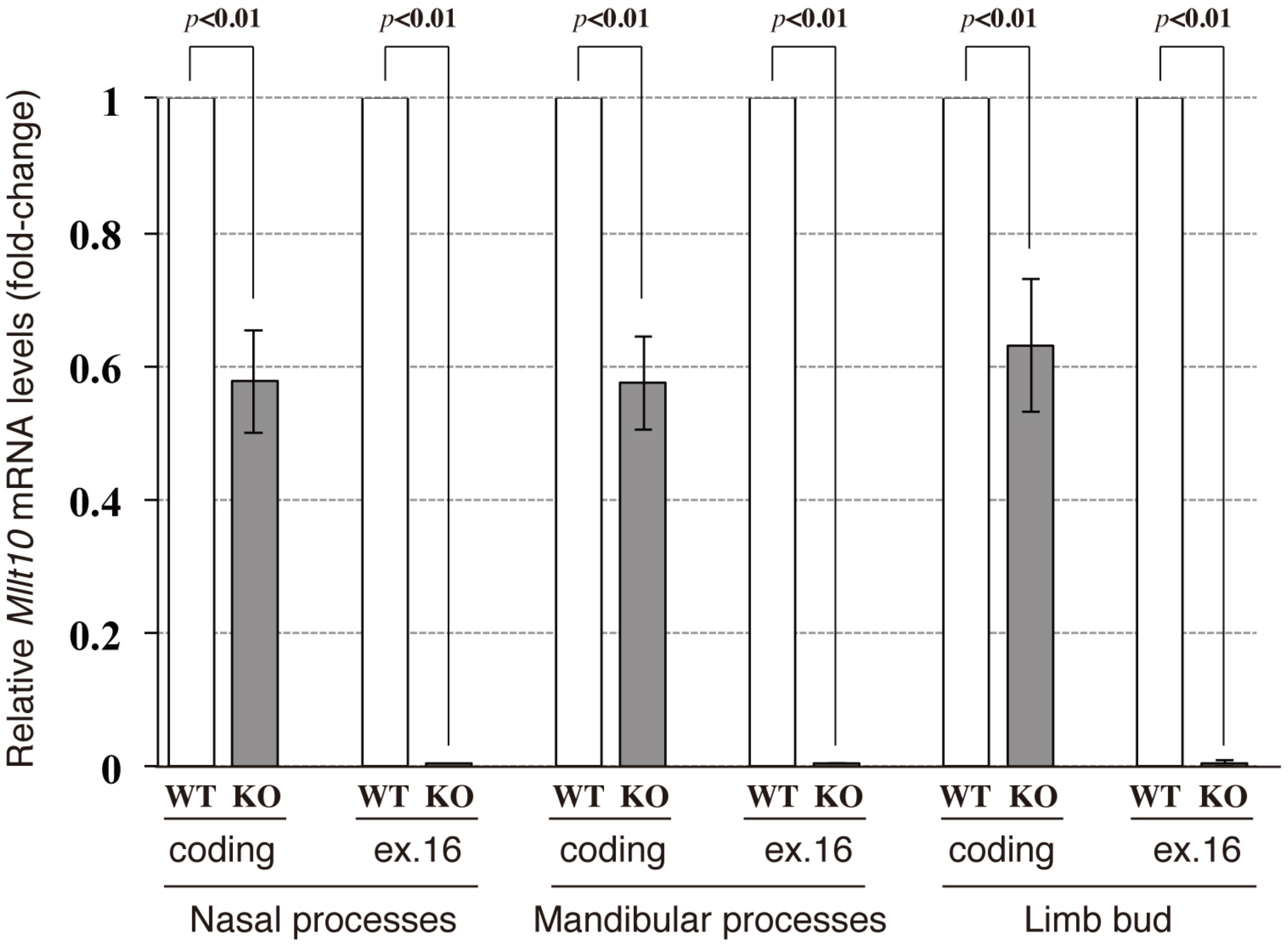

Supplementary Figure S9

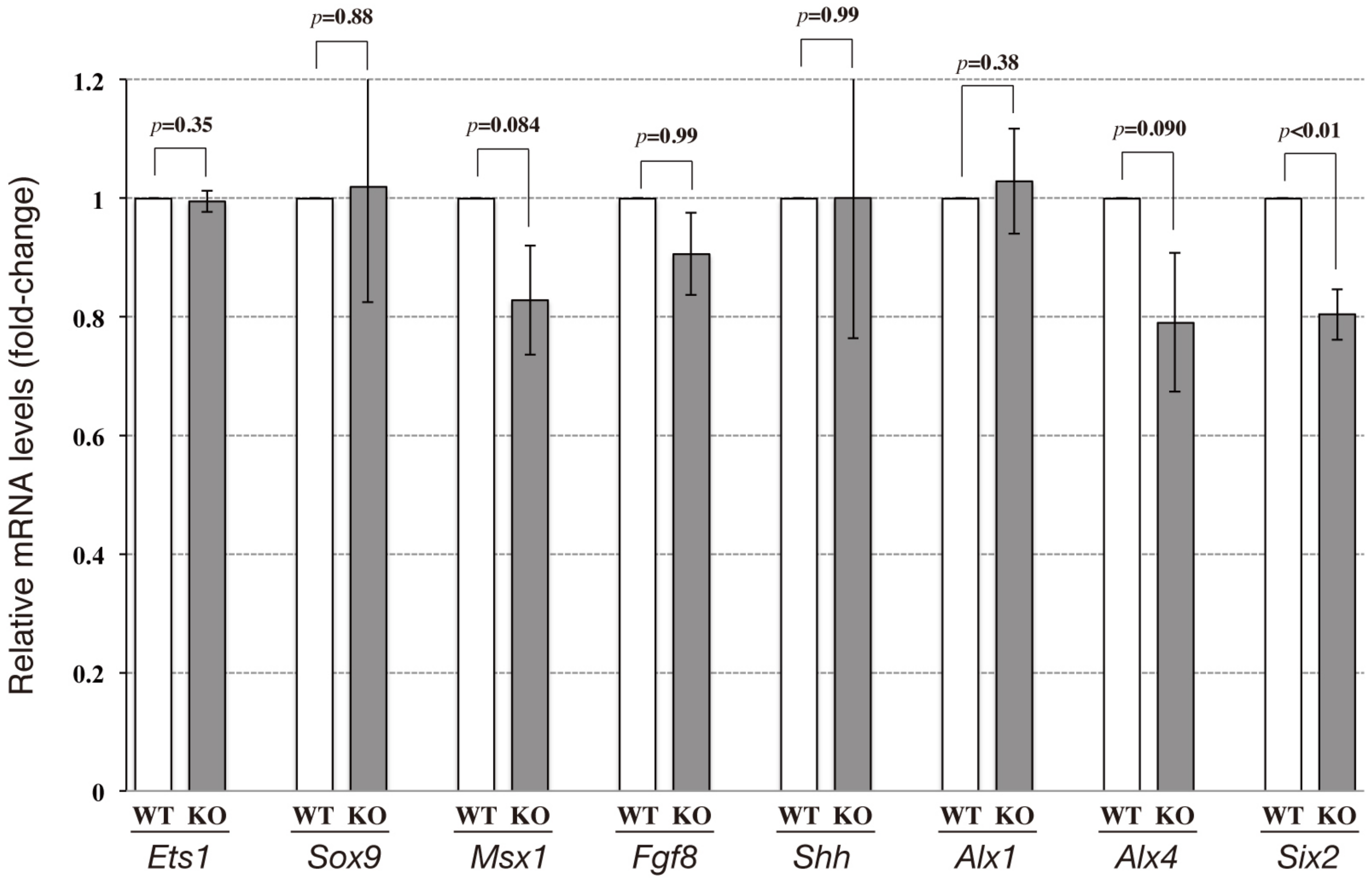

Supplementary Figure S10

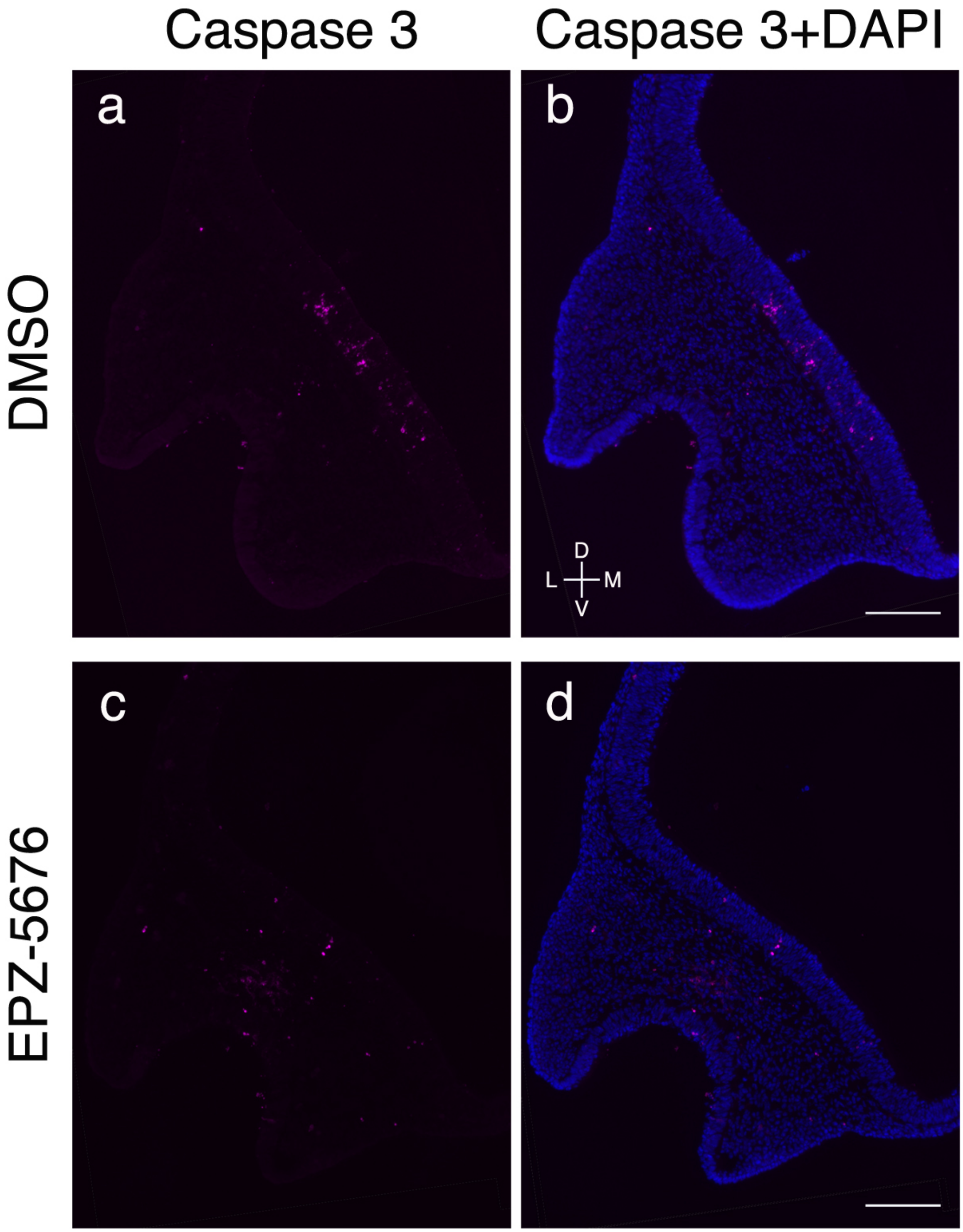

Original images

Fig.S1 f, g

LP SP

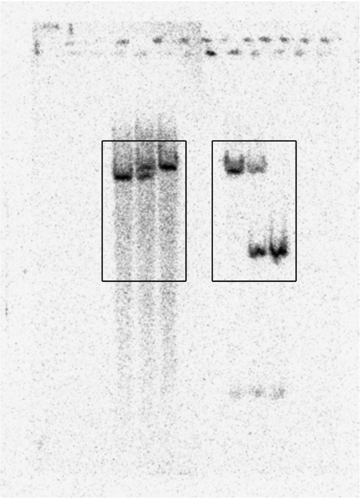

Fig.S1 h

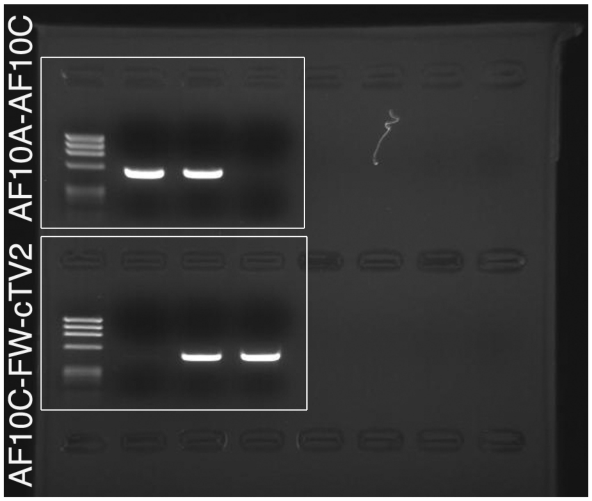

Fig.S1 i

anti-Af10 antibody anti-β actin antibody

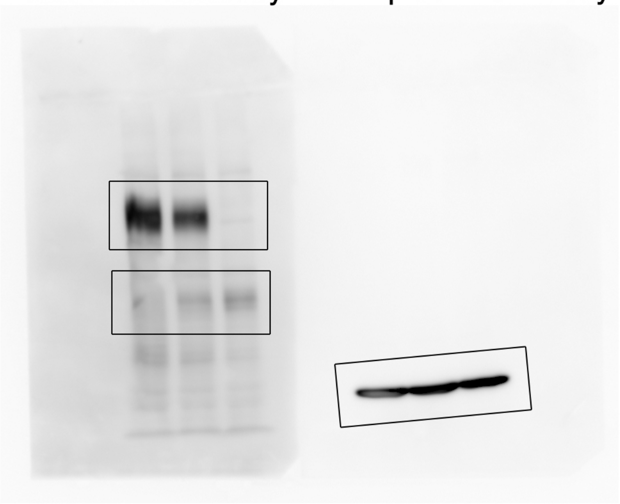

Fig.5 j

anti-H3K79me2 antibody

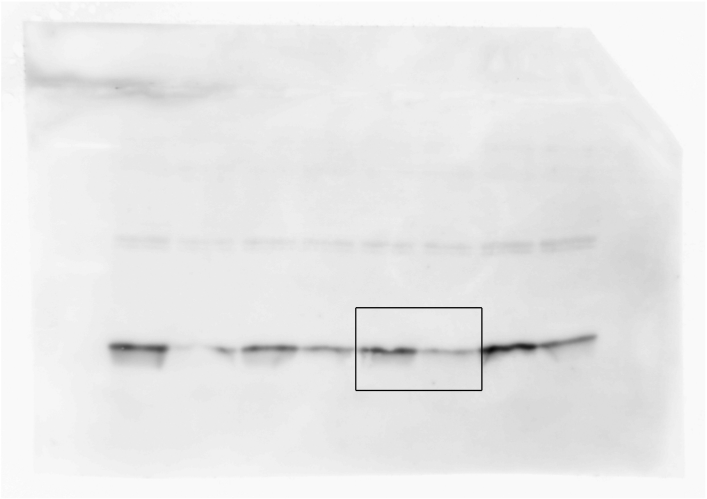

Fig.5 j

anti-histone H3 antibody

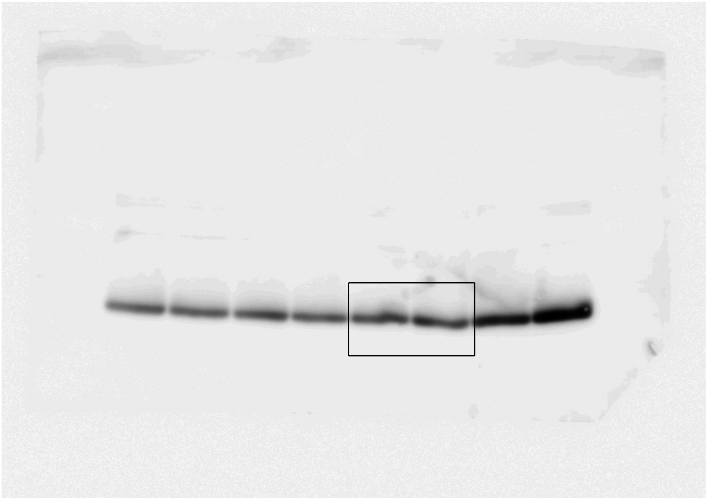

Supplement: Supplementary file 1 — Supplementary information [file 41598_2017_11745_MOESM1_ESM.pdf]
